# Comparisons of Inverse Dynamics Formulations in a Spatial Redundantly Actuated Parallel Mechanism Constrained by Two Point Contact Higher Kinematic Pairs

**DOI:** 10.3390/biomimetics9090564

**Published:** 2024-09-18

**Authors:** Chen Cheng, Xiaojing Yuan, Yenan Li, Jian Liu

**Affiliations:** Laboratory of Mechatronics, Xi’an College of Technology, Xi’an 710025, China

**Keywords:** inverse dynamics, dynamics formulation, higher kinematic pair, spatial parallel mechanism, redundant actuation

## Abstract

A spatial redundantly actuated parallel mechanism (RAPM) constrained by two point contact higher kinematic pairs (HKPs) has been designed, arising from the inspiration of mastication in human beings: the end effector is the lower jaw, the six kinematic chains are the primary chewing muscles, and the constraints at HKPs are the temporomandibular joints. In this paper, firstly, the constrained motions of the mechanism are described in detail; thereafter, five models are formulated by the well-known Newton–Euler’s law, the Lagrangian equations, and the principle of virtual work, to explore its rigid-body inverse dynamics. The symbolic results show that the model structures based on these approaches are quite different: the model via the Newton–Euler law well reflects the nature of the mechanism in terms of the constraint forces from HKPs with six equations and eight unknowns, and the existence of reaction forces at the spherical joints is tightly dependent on the number of kinematic chains. In comparison, from the latter two methods, the constraint forces and the reaction forces at spherical joints do not appear in the four models in which there are only four equations and six unknowns. Further, by using the dynamics model of the non-redundantly actuated counterpart as the core in both the second models from the energy and virtual work-related methods, their computational cost is only about 16.7% and 36.63% of the two first models, respectively. Finally, the comparisons between the dynamics models of the RAPM and its counterpart clarify that the HKP constraints greatly alter the model structures and raise the technical difficulties.

## 1. Introduction

In the human masticatory system, the mandible can perform masticatory behaviours in terms of motions and bite forces in the three-dimensional (3D) space, driven by the muscle contractions in different ways [1]. Simultaneously, it is always pivoted at the condyles via the left and right temporomandibular joints (TMJs), which are between the temporal bone of the skull and the mandibular condyles. From the viewpoint of mechanism, this system is inherently a spatial parallel mechanism (PM) with high actuation redundancies, since it has a greater number of mastication muscles than the degrees of freedom (DOFs) of the mandible. A spatial redundantly actuated parallel mechanism (RAPM) constrained by two higher kinematic pairs (HKPs) for mastication has been built in a bio-inspired manner, where the base is the skull, the six kinematic chains are the most primary chewing muscles, the end effector is the mandible, and the two HKPs are employed acting as TMJs at the two sides, respectively [2,3]. A comprehensive review of the biomechanical findings of the human jaw structure and chewing muscles, masticatory robotics, and their applications was reported in Chapter 1 of [4]. It is noted that though the target mechanism has been designed according to the human chewing system, we only study the inverse dynamics of this mechanism, rather than the biomechanics of the chewing system. Whether readers are familiar with this biological system or not does not influence their understanding of inverse dynamics of the robotic mechanism.

PMs are superior to their serial counterparts in terms of larger stiffness, larger dynamic load-carrying capacity, higher motion accuracy, and lower inertia; as such, they are extensively employed in a variety of domains where these merits are of great interest [5,6,7,8,9]. These advantages are further enhanced by actuation redundancy, which can eliminate singularities to expand the useful workspace, control antagonistic internal forces to alleviate backlashes, increase the energy efficiency, and raise the dynamic performance, etc., as shown in [10,11,12,13,14,15,16,17]. The state of the art of their extensive applications can be found in [18]. Generally, PMs with or without redundant actuations are only composed of lower kinematic pairs, as in these above-mentioned publications; in this paper, nevertheless, the RAPM under study is characterised by two HKP constraints which bring two redundant actuations, being innovative and rare in the mechanism design.

The RAPM with two HKPs at hand has been designed to evaluate the time-varying dynamics of food textures in a biomimetic fashion as a robotic device in the food industry. Accordingly, in its practical applications, it is necessary to precisely reproduce the chewing behaviours of human beings using this mechanism, in terms of the 3D chewing motions and bite forces. As such, an accurate and computationally efficient inverse dynamics model is fundamental to its mechanical design, performance evaluation, and real-time motion and/or force control.

The classical techniques including Newton–Euler’s law, the Lagrangian formulation, and the principle of virtual work proposed for general mechanical systems are broadly adopted to find the inverse dynamics solutions of RAPMs. The dynamics of a planar 3-DOF RAPM was analysed based on Newton–Euler’s law in [19]. By utilising the left-right symmetry of the manipulator, the law was applied to the left and the right bodies, and the end effector, respectively, to obtain a simplified dynamics model. The maximum dynamic load-carrying capacity of a planar 3-DOF RAPM was analysed also via Newton–Euler’s law in [20]. In [21], the inverse dynamics of the 4RRR planar RAPM was solved to achieve the shaking force/moment balancing. The underlined letter indicates that it is the active joint in the mechanism throughout this paper.

In [22], the Lagrange formulation with unknown Lagrangian multipliers was used to address the dynamics of the 2RRR/RR RAPM. The multipliers representing the magnitude of constraint forces were eliminated using the null space of the differential matrix of the closed-loop constrained equation. The identical procedure was also utilised in a planar 2-DOF RAPM with parallelograms in [23]. In [24], firstly, Newton–Euler’s law was applied to derive the dynamics of the chain of a variable mass 3RRR planar RAPM; then, the Lagrange multipliers technique was used to establish the dynamics model of the entire mechanism. The classical partitioning method was applied to compute the multipliers. The Lagrangian equations of the second type without Lagrange multipliers were employed to solve the inverse dynamics of a 5UPS/PRPU RAPM with five DOFs in [25]. In [14], the dynamics model was formatted for a 4PUS/PPPU RAPM with five DOFs and seven actuators using the Lagrange method first in the task space. Then, by virtue of the generalized left inverse of the Jacobian matrix mapping the velocity of the end effector into that of the active joints, this model was ultimately built in the joint space. To minimise the energy consumption of a planar PM, redundant actuations were employed in [26]. The dynamics model was established using the Lagrange method. With the same method, the dynamics equations of a 3-DOF spatial RAPM were written in [27]. A simplified inverse dynamic method based on the Lagrangian approach was proposed in [28], where the key concept was simplifying the expression of the energy.

In [29], the principle of virtual work was used to build the model of the same planar 3-DOF RAPM from [19], and then a position and force switching control strategy was designed. In [30], this principle was employed to solve the inverse dynamics of an 8PSS spatial RAPM. The principle of virtual work was used to derive the dynamics model of a 4PSS/PU spatial RAPM with three DOFs in [31], to measure the maximum angular and translational accelerations, respectively. In [32], to evaluate the dynamics performance of planar PMs with actuation and kinematic redundancies, firstly, Newton–Euler’s law was used to determine the resultant wrenches acting at each body. Then, the principle of virtual work was used to find the input torques. In [17], this principle expressed by generalised coordinates was used to establish the dynamics model of RAPMs. A 6PUS + UPU RAPM with 5 DOFs was employed as a case study. This principle and the screw theory were together employed in a 4PPPS RAPM with twelve actuators and six redundant actuations in [16], to address the high redundancy efficiently. The principle of virtual work was employed in [33] to propose a new torque optimisation method for a 3-DOF RAPM.

From this short review, it is remarked that the inverse dynamics solution is mainly about RAPMs consisting of lower kinematic pairs. In our previous work [3], an initial attempt at the rigid body dynamics of the RAPM has been made via the hybrid of the Lagrangian equations and Newton–Euler’s law, which is quite complex and error-prone: the forces at HKPs without friction effects are ideal constraints which must not be included in the Lagrangian formulation, whilst they must be considered under Newton–Euler’s law. In fact, the model can be established by the two formulations independently and separately, which is the first motivation for writing this paper. Meanwhile, the influence of the HKP constraints on the modelling process, the final model structure, the numerical results, and the computational cost has not been figured out clearly, which is the second motivation for carrying out the related study.

In this paper, it is assumed that all the bodies including the HKP constraints and rotational and spherical joints are rigid, frictionless, and free of clearances. The inertia of the spherical joints is quite small and then is not considered in the formulation. The sequel begins with a detailed description of the mechanism. Next, the constrained motion of the end effector and the kinematics of the chains are derived. Thereafter, five dynamics models are built analytically from Newton–Euler’s law, the Lagrangian formulation, and the principle of virtual work. Finally, numerical computations are conducted to verify and compare the correctness and computational efficiency of the models. The role of HKP constraints in the modelling is not only investigated by the two models from the latter two methods, respectively, but also comparatively examined with the 6RSS PM in Chapter 4 of [4], which does not have HKPs.

The main contributions of this paper are:

1. The inverse dynamics solution of the constrained RAPM has been explored deeply via Newton–Euler’s law, the Lagrangian formulation, and the principle of virtual work, respectively;
2. Under the latter two methods, using the dynamics model of the 6RSS PM as the core of the RAPM’s model can considerably alleviate the computational demands;
3. The insight into the influence of HKP constraints on the inverse dynamics has been provided clearly in terms of the model structure, the numerical results, and the computational cost.

## 2. The Robotic Mechanism

**Figure 1:**
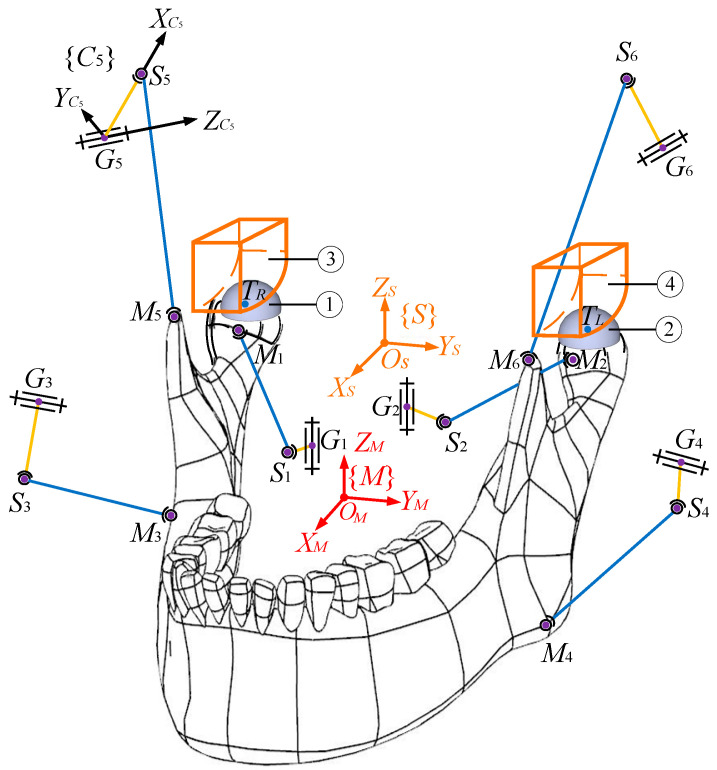
Kinematic diagram of the RAPM constrained by point-contact HKPs, where ➀ right condyle ball, ➁ left condyle ball, ➂ articular surface of right TMJ, ➃ articular surface of left TMJ [2].

The kinematic diagram of the RAPM constrained by two HKPs is illustrated in Figure 1. The maxilla (i.e., the base) is fixed on the ground and the movable mandible (i.e., the end effector) is connected to the base by six independent kinematic chains. The maxilla, to which the inertia frame {*S*} is assigned, is not shown in the figure for a clear exhibition of movable bodies. This frame consists of a horizontal *X_S_*-*Y_S_* plane perpendicular to the vertical *Z_S_* axis. A frame {*M*} is established at the mass centre *O_M_* of the end effector. The origins and orientations of {*S*} and {*M*} overlap when the mechanism is at the home position, that is, the maxilla and the mandible are in the occlusal state. The origin *O_M_* is used as the reference point to describe the mandibular translations, and its orientations with respect to {*S*} are described by *XYZ* Euler angles, that is, α,β and γ around the three axes of {*M*}. The layout of the six chains is in accordance with the six primary masticatory muscles of human beings. Each chain contains a rotational actuator fixed onto the base, whose driving shaft connects a crank ***G****_i_****S****_i_* (*i* = 1, …, 6) with a rotational joint at *G_i_*, and a coupler ***S****_i_****M****_i_* that joins the crank and the end effector via two spherical joints at its two ends *S_i_* and *M_i_*, respectively. The rotation of the *i*th actuator with respect to {*S*} is described by the actuator frame $\left\{C_{i}\right\}$ attached at *G_i_*. In it, the $X_{C_{i}}$ axis is directed from *G_i_* to *S_i_*, the $Z_{C_{i}}$ axis runs through the driving shaft of the actuator, and the $Y_{C_{i}}$ axis completes the frame, obeying the right-hand rule. A frame {*N_i_*} is attached at the mass centre *E_i_* of ***S****_i_****M****_i_* to describe its motions with respect to {*S*}. The $X_{N_{i}}$ axis points from *S_i_* to *M_i_*, the $Y_{N_{i}}$ axis is parallel to the cross product of two unit vectors defined along the $X_{N_{i}}$ and *X_S_* axes, and the $Z_{N_{i}}$ axis is defined by the right-hand rule.

**Figure 2:**
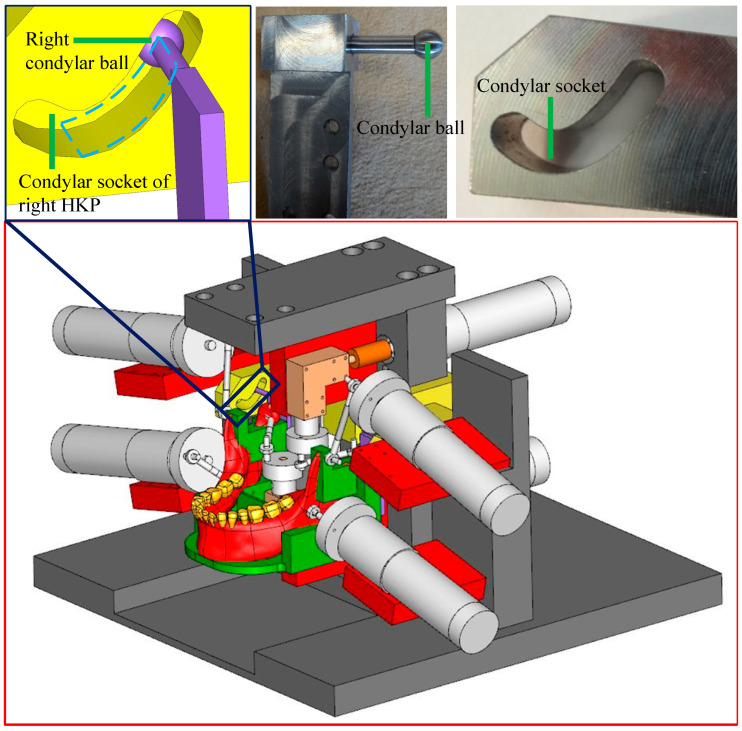
CAD model of the RAPM, magnification of the right HKP and its prototype [3].

Two HKPs modelling the left and right TMJs are formed by the two condyle balls being in contact with the articular surfaces. In the mechanism prototype, the two contacts between the left and right condyles of the mandible and the maxilla at TMJs are realised by the two condyle balls being always constrained within a curved condylar socket, as shown in Figure 2. The width of the socket equals the diameter of the condyle ball; thus, it can always guarantee the point contact during the movement of the mandible. By this design, the motion of the condyle ball centre is always constrained onto a surface, which is offset from the upper and lower surfaces of the socket by the ball radius. Thereupon, it is clear that the end effector is actuated by six chains and constrained by the environment at the two HKPs simultaneously.

## 3. Kinematics of the Mechanism

### 3.1. Constrained Motions of the End Effector

A second-order surface has been designed according to [34], being used as the workspace of the centre of the condylar ball as in [2,3]. Its cross-section is identical along the *Y_S_* axis in {*S*}, being in accordance with that in [1], and the range of the lateral movement of the lower jaw has been determined from [35]. However, on the one hand, when the mechanism tracks the real chewing trajectories of healthy human subjects, the condylar ball mainly slips along the curved surface surrounded by blue dashed lines as in Figure 2, which can be easily approximated by a flat one. On the other hand, it is quite difficult to derive the analytical expressions of the parasitic motions in the RAPM under the second-order surface. In this regard, in this paper where the chewing system is explored from the viewpoint of mechanical dynamics and with an emphasis on the constrained dynamics of the end effector, the surfaces in {*S*} where the left and right condyle ball centres *T_L_* and *T_R_* slide on are designed as flat (unit: mm):

(1) $$\begin{array}{l} \begin{array}{ccc} Z_{L}=p_{1}X_{L}+p_{2}, & p_{3}\leq X_{L}\leq p_{4}, & \;\;\;p_{5}\leq Y_{L}\leq p_{6} \end{array}\\ \begin{array}{ccc} Z_{R}=p_{1}X_{R}+p_{2}, & p_{3}\leq X_{R}\leq p_{4}, & -p_{6}\leq Y_{R}\leq -p_{5} \end{array}\\ p_{1}=-1.1,\;p_{2}=13.215,\;p_{3}=-27.65,\;p_{4}=-14.65,\;p_{5}=69,\;p_{6}=75 \end{array}$$

From the Kutzbach–Grübler criterion, the mechanism now has four DOFs, but the information on which four DOFs to choose is not given. They are to be derived from a rigorous computation below: the coordinates of *T_i_* (*i* = *L*, *R*) in {*S*} can be expressed as

(2) OSTi=XiYiZi=OSOM+RMS⋅OMMTi

where $O_{S}O_{M}={\left[\begin{array}{ccc} X & Y & Z \end{array}\right]}^{T}$ denotes the 3×1 position vector of *O_M_* in {*S*}, RMS=RXα⋅RYβ⋅RZγ is the rotation matrix from {*S*} to {*M*}, $R_{X}\left(\alpha \right),R_{Y}\left(\beta \right)$, and $R_{Z}\left(\gamma \right)$ are three rotation matrices about the *X_M_*, *Y_M_*, and *Z_M_* axes by α,β and γ, respectively. It is worth noting that in this paper, a matrix/vector/scalar in local frames owns a leading superscript on its left to denote the specific frame it refers to, but those in {*S*} omit their superscripts for the sake of convenience and clarity. The six motion variables of the end effector are grouped and expressed as

(3) $$X_{EE}={\left[\begin{array}{cccccc} X & Y & Z & \alpha & \beta & \gamma \end{array}\right]}^{T}$$

From Equation (2), one can obtain

(4) XL=X+R(1,:)MS⋅OMMTL,XR=X+R(1,:)MS⋅OMMTRZL=Z+R(3,:)MS⋅OMMTL,ZR=Z+R(3,:)MS⋅OMMTR

where Ri,:MS is the *i*th (*i* = 1, 3) row of RMS. Putting Equation (4) into Equation (1) produces

(5) Z+R(3,:)MS⋅OMMTL=p1⋅X+R(1,:)MS⋅OMMTL+p2Z+R(3,:)MS⋅OMMTR=p1⋅X+R(1,:)MS⋅OMMTR+p2

In view of the left-right symmetry of OMMTL and OMMTR in {*M*}, a summation and a subtraction of the two equations in Equation (5) sidewise yield

(6) Z=p1X+p2+p1⋅R(1,:)MS−R(3,:)MS⋅OMMTL10OMMTL3γ=−atansαp1cβ+cαsβ

where OMMTL1 and OMMTL3 are the first and third terms of OMMTL, respectively, and the two letters *c* and *s* are short for cos and sin, respectively. From these computations, it is found *Z* and γ are transferred from DOFs to parasitic motions and they are functions of $q_{EE}$, which is a 4×1 vector by grouping four DOFs as

(7) $$q_{EE}={\left[\begin{array}{cccc} X & Y & \alpha & \beta \end{array}\right]}^{T}$$

It constitutes the task space of the mechanism. To characterise the instantaneous configuration of the mechanism, Equation (3), or both Equations (6) and (7) ad hoc are needed. In other words, it can still perform motions in six directions with four DOFs and two parasitic motion variables. Regarding this, redundant actuations in the mechanism are essentially caused by constraints from the base directly onto the end effector, which is completely different from the two methodologies mentioned in [11]. It is also worth noting that though the workspace of the centre of the condylar ball is simplified as a flat surface as in Equation (1), there is still a strongly nonlinear and sophisticated relationship between *Z*/γ and ***q****_EE_* in Equation (6).

After figuring out the DOFs of the end effector, its motions can be defined below. The angular velocity is

(8) $$\omega_{EE}=M_{0b}\cdot {\dot{q}}_{EE}$$

where

$$\begin{array}{l} M_{0b}=M_{0a}\cdot M_{J}\\ M_{0a}=\left[\begin{array}{cc} 0_{3} & R_{\omega } \end{array}\right]\\ R_{\omega }=\left[\begin{array}{ccc} 1 & 0 & s\beta \\ 0 & c\alpha & -s\alpha c\beta \\ 0 & s\alpha & -c\alpha c\beta \end{array}\right] \end{array}$$

$0_{3}$ is the 3×3 zero matrix, and ***M****_J_* denotes the 6×4 Jacobian matrix between ***X****_EE_* and ***q****_EE_*, namely

(9) $$\begin{array}{l} M_{J}=\mathrm{Jacobian}\left(\begin{array}{cc} X_{EE}, & q_{EE} \end{array}\right)\\ {\dot{X}}_{EE}=M_{J}\cdot {\dot{q}}_{EE} \end{array}$$

The translational velocity of the end effector is expressed by

(10) $$V_{O_{M}}=M_{1b}\cdot {\dot{q}}_{EE}$$

where $M_{1b}=M_{1a}\cdot M_{J},M_{1a}=\left[\begin{array}{cc} E_{3} & 0_{3} \end{array}\right]$, and ***E***_3_ is the 3×3 identity matrix. The angular and translational accelerations of the end effector are found by the differentiation of Equations (8) and (10) with respect to time, respectively:

(11) $$\begin{array}{l} {\dot{\omega }}_{EE}={\dot{M}}_{0b}\cdot {\dot{q}}_{EE}+M_{0b}\cdot {\ddot{q}}_{EE}\\ {\dot{V}}_{O_{M}}={\dot{M}}_{1b}\cdot {\dot{q}}_{EE}+M_{1b}\cdot {\ddot{q}}_{EE} \end{array}$$

### 3.2. Kinematics of the ith Chain

The inverse kinematics of the mechanism, i.e., $\theta =\theta \left(q_{EE}\right)\left(\theta ={\left[\begin{array}{ccc} \theta_{1} & \cdots & \theta_{6} \end{array}\right]}^{T}\right)$ that constitutes a system of six decoupled equations expressed by $q_{EE}$, has already been derived in Section 3.2 of [36]. Nevertheless, the motions of the coupler ***S****_i_****M****_i_* (*i* = 1, …, 6) are still needed for its rigid-body dynamics. Due to the two spherical joints at ***S****_i_* and ***M****_i_*, the coupler can rotate around the three orthogonal axes of {*N_i_*}. The rotation around $X_{N_{i}}$ axis is a passive DOF for it is not controllable; this rotational range is quite small thanks to the physical restrictions from the used spherical joints in the mechanical design, however. At these regards, it is assumed that there is no axial rotation in the coupler. Two Euler angles $\beta_{i}$ and $\gamma_{i}$ around the $Y_{N_{i}}$ and $Z_{N_{i}}$ axes, respectively, are used to express the rotation of ***S****_i_****M****_i_* in {*S*} in terms of the two rotational matrices $R_{Y}\left(\beta_{i}\right)$ and $R_{Z}\left(\gamma_{i}\right)$. Thereafter, the coordinate vector of the coupler can be expressed as

(12) SiMi=RNi0S⋅RYβi⋅RZγi⋅SiMi00

where RNi0S is the orientation of ***S****_i_**M**_i_* in {*S*} at the initial configuration of the mechanism at hand, and $\left\|S_{i}M_{i}\right\|$ is the length of ***S****_i_**M**_i_*. From the geometry of the mechanism in {*S*}, the vector of the coupler can be found from the difference in the position vector of *M_i_* and *S_i_*:

(13) $$S_{i}M_{i}=O_{S}O_{M}+O_{M}M_{i}-O_{S}G_{i}-G_{i}S_{i}$$

where

OMMi=RMS⋅OMMMi and OMMMi is the coordinate vector of *M_i_* in {*M*};

$O_{S}G_{i}$ is the constant position vector of *G_i_* in {*S*};

And GiSi=RCi0S⋅RZθi⋅GiSi00, in which RCi0S is the orientation of ***G****_i_****S****_i_* in {*S*} at the initial configuration of the mechanism, $R_{Z}\left(\theta_{i}\right)$ is the rotation matrix about the $Z_{C_{i}}$ axis by $\theta_{i}$, and $\left\|G_{i}S_{i}\right\|$ is the length of $G_{i}S_{i}$.

Substituting Equation (12) and $\theta_{i}=\theta_{i}\left(q_{EE}\right)$ into Equation (13) produces

(14) $$\begin{array}{l} \beta_{i}=-a\tan\frac{n_{i3}}{n_{i1}}\\ \gamma_{i}=a\sin n_{i2} \end{array}$$

where ni1ni2ni3=R−1Ni0S⋅SiMili. For the sake of convenience, a 3×1 generalised vector defined as $q_{r_{i}}=\left[\begin{array}{c} \theta_{i}\\ \beta_{i}\\ \gamma_{i} \end{array}\right]$ consists of the joint space of the *i*th chain and it is adopted to completely specify its configuration. It is highlighted that $q_{r_{i}}$ is the function of $q_{EE}$, i.e., $q_{r_{i}}=q_{r_{i}}\left(q_{EE}\right)$.

To derive the relationship between the first-time derivative of $q_{r_{i}}$ and $q_{EE}$, Equation (13) can be rewritten as

(15) $$O_{S}G_{i}+G_{i}S_{i}+S_{i}M_{i}=O_{S}O_{M}+O_{M}M_{i}$$

The left- and right-hand sides can be expressed by $q_{r_{i}}$ and $q_{EE}$, respectively. The first-time derivative of Equation (15) yields

(16) $$M_{1i}\cdot {\dot{q}}_{r_{i}}=M_{2i}\cdot {\dot{q}}_{EE}$$

where

$$\begin{array}{l} M_{1i}=\mathrm{Jacobian}\begin{array}{cc} (O_{S}G_{i}+G_{i}S_{i}+S_{i}M_{i}, & q_{r_{i}}) \end{array}\\ M_{2i}=\mathrm{Jacobian}\begin{array}{cc} (O_{S}O_{M}+O_{M}M_{i}, & q_{EE}) \end{array} \end{array}$$

Moreover, one can find that

(17) $${\dot{q}}_{r_{i}}=M_{3i}\cdot {\dot{q}}_{EE}$$

where $M_{3i}=M_{1i}^{-1}\cdot M_{2i}$, and differentiating Equation (16) produces that

(18) $${\dot{M}}_{1i}\cdot {\dot{q}}_{r_{i}}+M_{1i}\cdot {\ddot{q}}_{r_{i}}={\dot{M}}_{2i}\cdot {\dot{q}}_{EE}+M_{2i}\cdot {\ddot{q}}_{EE}$$

Upon substitution of Equation (17) into Equation (18), it gives rise to the second-time derivative of $q_{r_{i}}$ as

(19) $${\ddot{q}}_{r_{i}}=M_{3i}\cdot {\ddot{q}}_{EE}+M_{4i}\cdot {\dot{q}}_{EE}$$

where $M_{4i}=M_{1i}^{-1}\cdot \left({\dot{M}}_{2i}-{\dot{M}}_{1i}\cdot M_{3i}\right)$. So far, ${\dot{q}}_{r_{i}}$ and ${\ddot{q}}_{r_{i}}$ have been derived as quantities intimately associated with the motion of the *i*th chain, rather than merely with its configuration.

The rotational velocity of ***S****_i_****M****_i_* is

(20) ωSiMi=RNi0S⋅RωSiMi⋅q˙ri=RNi0S⋅RωSiMi⋅M3i⋅q˙EE

where $R_{\omega S_{i}M_{i}}=\left[\begin{array}{cc} \begin{array}{c} 0\\ 0\\ 0 \end{array} & \begin{array}{cc} 0 & s\beta_{i}\\ 1 & 0\\ 0 & c\beta_{i} \end{array} \end{array}\right]$, and its rotational acceleration is

(21a) ω˙SiMi=RNi0S⋅∂RωSiMi∂qriT⋅q˙ri⊗E3⋅q˙ri+RωSiMi⋅q¨ri

Putting Equations (17) and (19) into Equation (21a), ${\dot{\omega }}_{S_{i}M_{i}}$ can also be expressed by $q_{EE},{\dot{q}}_{EE},{\ddot{q}}_{EE}$ as

(21b) ω˙SiMi=RNi0S⋅∂RωSiMi∂qriT⋅M3i⋅q˙EE⊗E3⋅M3i⋅q˙EE+RωSiMi⋅M3i⋅q¨EE+M4i⋅q˙EE

which is more complicated with more symbols and arithmetic operations than Equation (21a).

The coordinate vector of the mass centre *E_i_* in {*S*} is

(22) OSEi=OSGi+GiSi+SiEi=OSGi+RCi0S⋅RZθi⋅GiSi00+RNi0S⋅RYβi⋅RZγi⋅0.5⋅SiMi00

The translational velocity and acceleration of *E_i_* can then be found by the differentiation of Equation (22) with respect to time once and twice, respectively

(23) $$\begin{array}{l} V_{E_{i}}=J_{E_{i}}\cdot {\dot{q}}_{r_{i}}\\ {\dot{V}}_{E_{i}}={\dot{J}}_{E_{i}}\cdot {\dot{q}}_{r_{i}}+J_{E_{i}}\cdot {\ddot{q}}_{r_{i}} \end{array}$$

where

$$\begin{array}{l} J_{E_{i}}=\mathrm{Jacobian}\left(\begin{array}{cc} O_{S}E_{i}, & q_{r_{i}} \end{array}\right)\\ {\dot{J}}_{E_{i}}=\frac{\partial J_{E_{i}}}{\partial q_{r_{i}}^{T}}\cdot \left({\dot{q}}_{r_{i}}⊗E_{3}\right) \end{array}$$

Upon substitution of Equations (17) and (19) into Equation (23), it yields

(24) $$\begin{array}{l} V_{E_{i}}=J_{E_{i}}\cdot M_{3i}\cdot {\dot{q}}_{EE}\\ {\dot{V}}_{E_{i}}=\left({\dot{J}}_{E_{i}}\cdot M_{3i}+J_{E_{i}}\cdot M_{4i}\right)\cdot {\dot{q}}_{EE}+J_{E_{i}}\cdot M_{3i}\cdot {\ddot{q}}_{EE} \end{array}$$

With this kinematics background provided so far in the mechanism of interest, one is in a position to write equations of motion based on the three inverse dynamics methods below.

## 4. Newton–Euler Formulation

The Newton–Euler law is first applied to each body including the end effector, the coupler and the crank in each chain. The equations of motion are subsequently ordered suitably, arriving at the dynamics model of the entire mechanism at hand.

### 4.1. The End Effector

**Figure 3:**
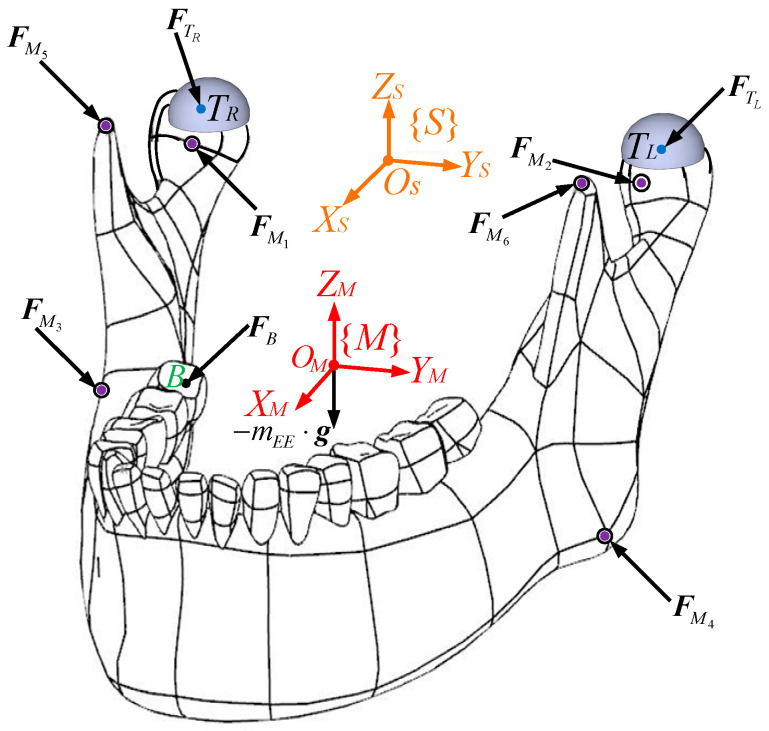
Free-body diagram of the end effector.

The free-body diagram of the end effector is shown in Figure 3. The forces acting on it include:

the constrained forces $F_{T_{j}}\left(j=L,R\right)$ at the left and right condylar balls, respectively;

the six reaction forces $F_{M_{i}}\left(i=1,\ldots ,6\right)$ which are exerted from the six couplers;

its gravitational force $-m_{EE}\cdot g$ at the mass centre *O_M_*, in which *m_EE_* is its mass and $g=\begin{array}{ccc} {}[0 & 0 & 9800] \end{array}mm/s^{2}$ is the gravitational acceleration vector;

and the bite force $F_{B}$ at the point *B* on the right molar which is predefined in inverse dynamics.

**Figure 4:**
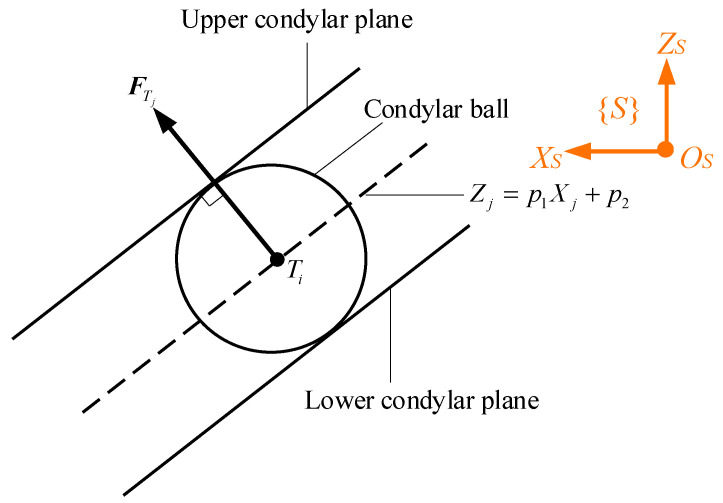
Sagittal view of the condylar ball and the constrained force.

Thanks to the specific design for the workspace of the condylar ball centres, $F_{T_{j}}\left(j=L,R\right)$ can be expressed as

(25) $$F_{T_{j}}=M_{H}\cdot F_{Z_{j}}$$

where $M_{H}={\left[\begin{array}{ccc} -p_{1} & 0 & 1 \end{array}\right]}^{T}$, and $F_{Z_{j}}\left(j=L,R\right)$ is the unknown magnitude of the constrained force at HKPs along *Z_S_* axis. If $F_{Z_{j}}$ is positive/negative, it means that the condylar ball is receiving constrained forces from the lower/upper surface of the condylar socket. The sagittal view of the condylar ball and the constrained force are given in Figure 4, indicating the flat lower condylar surface is exerting a constraint force onto the condylar ball at that time instant.

The Newton–Euler law of the end effector gives

(26) $$M_{2b}\cdot F_{M_{1\_6}}=M_{3b}-M_{4b}\cdot F_{Z}$$

where $F_{M_{1\_6}}={\left[\begin{array}{c} F_{M_{1}}\\ \vdots \\ F_{M_{6}} \end{array}\right]}_{18\times 1},F_{Z}=\left[\begin{array}{c} F_{Z_{L}}\\ F_{Z_{R}} \end{array}\right]$, and the detailed descriptions of other variables are presented in the Appendix A, for a better understanding of the concept.

### 4.2. The Coupler S_i_M_i_

**Figure 5:**
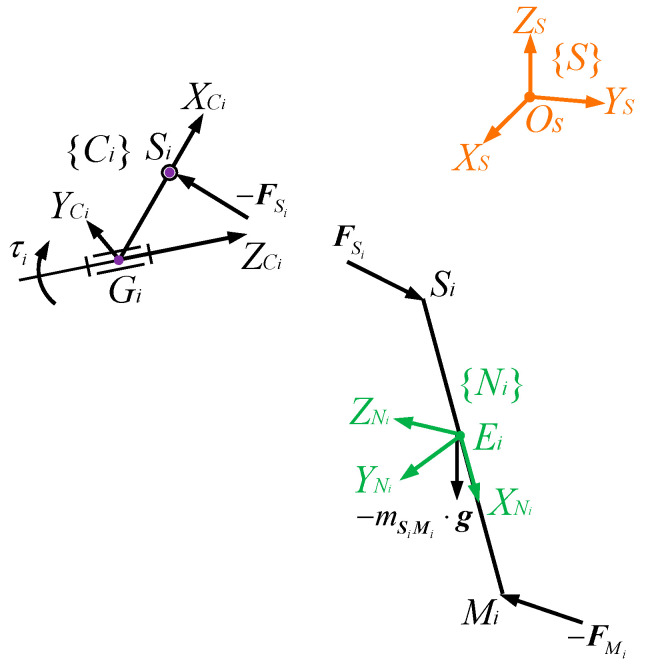
Free-body diagram of the crank and the coupler in the *i*th chain.

The force acting at the *i*th coupler ***S****_i_****M****_i_* are shown in Figure 5, and its Newton–Euler formulation is

(27) $$\begin{array}{l} F_{S_{i}}-F_{M_{i}}=m_{S_{i}M_{i}}\cdot {\dot{V}}_{E_{i}}+m_{S_{i}M_{i}}\cdot g\\ E_{i}S_{i}\times F_{S_{i}}+E_{i}M_{i}\times \left(-F_{M_{i}}\right)=I_{S_{i}M_{i}}\cdot {\dot{\omega }}_{S_{i}M_{i}}+\omega_{S_{i}M_{i}}\times \left(I_{S_{i}M_{i}}\cdot \omega_{S_{i}M_{i}}\right) \end{array}$$

where $m_{S_{i}M_{i}}$ is the mass of ***S****_i_****M****_i_*, and $I_{S_{i}M_{i}}$ is the inertia tensor of ***S****_i_**M**_i_* with respect to its mass centre *E_i_*.

Combining the two equations in Equation (27) yields

(28) $$M_{i}S_{i}\times F_{M_{i}}=E_{S_{i}M_{i}}$$

Among the three equations in Equation (28), arbitrarily only two are independent. The first two are chosen for the following computation. For the six couplers, one can write that

(29) $$M_{5b}\cdot F_{M_{1\_6}}=M_{6b}$$

For the six couplers, from Newton’s equation in Equation (27), it can also be obtained that

(30) $$F_{S_{1\_6}}=F_{M_{1\_6}}+M_{7b}$$

where $F_{S_{1\_6}}={\left[\begin{array}{c} F_{S_{1}}\\ \vdots \\ F_{S_{6}} \end{array}\right]}_{18\times 1}$.

### 4.3. The Crank G_i_S_i_

The crank ***G****_i_**S**_i_* can only rotate around the $Z_{C_{i}}$ axis of frame $\left\{C_{i}\right\}$. For the sake of convenience, its one-dimensional rigid body dynamics is analysed in frame {*C_i_*_0_}, which is $\left\{C_{i}\right\}$ at the initial configuration of the mechanism. From Figure 5, the equation of motion of ***G****_i_**S**_i_* is

(31) τi+GiCi0Si×3,:⋅FSiCi0=IGiSi⋅θ¨i

where $\tau_{i}$ is the torque offered by the *i*th actuator, GiCi0Si is the 3×1 vector of $G_{i}S_{i}$ in {*C_i_*_0_}, GiCi0Si×3,: is the third line of the skew matrix GiCi0Si×, FSiCi0 is the reaction force exerted by the coupler at the crank and measured in {*C_i_*_0_}, and $I_{G_{i}S_{i}}$ is the rotational inertia of ***G****_i_****S****_i_* that is a scalar. GiCi0Si and FSiCi0 can be computed as

(32) GiCi0Si=RZθi⋅GiSi00FSiCi0=RTCi0S⋅−FSi

By substituting Equation (32) into Equation (31), it can be rewritten as

(33) $$\tau_{i}=M_{8bi}\cdot F_{S_{i}}+I_{G_{i}S_{i}}\cdot {\ddot{\theta }}_{i}$$

For the six cranks

(34) $$\tau =M_{8b}\cdot F_{S_{1\_6}}+M_{9b}$$

where $\tau =\left[\begin{array}{c} \tau_{1}\\ \vdots \\ \tau_{6} \end{array}\right]$.

### 4.4. The Entire Mechanism

Combining Equations (26) and (29), i.e., the Newton–Euler formulation of the end effector and the six couplers produces

(35) $${\left[\begin{array}{c} M_{2b}\\ M_{5b} \end{array}\right]}_{18\times 18}\cdot F_{M_{1\_6}}={\left[\begin{array}{c} M_{3b}\\ M_{6b} \end{array}\right]}_{18\times 1}-{\left[\begin{array}{c} M_{4b}\\ 0_{12\times 2} \end{array}\right]}_{18\times 2}\cdot F_{Z}$$

The constraint forces at *M_i_* can be computed as

(36) $$F_{M_{1\_6}}=M_{10b}-M_{11b}\cdot F_{Z}$$

Putting it into Equation (30) yields

(37) $$F_{S_{1\_6}}=M_{12b}-M_{11b}\cdot F_{Z}$$

Substituting Equation (37) into Equation (34) produces the dynamics model sought as

(38) $$\tau =M_{13b}-M_{14b}\cdot F_{Z}$$

It is shown that the inverse dynamics problem of the mechanism at hand can be reduced to solving a system of six linear equations in eight unknowns, which means Equation (38) is undetermined. This phenomenon is engendered by the constraints from HKPs to the end effector, which introduces two unknown constraint forces/redundant actuations/parasitic motions and eliminates two DOFs simultaneously.

## 5. Lagrangian Formulation

In writing the Lagrange equations of the RAPM, it must be emphasised that $F_{T_{L}}$ and $F_{T_{R}}$ are ideal constraint forces, as such, they do not appear in the formulation. There are two methods to build the models in this section: in Model 1, the equations are directly formatted to the RAPM; while in Model 2, intuitively, the dynamics model of the 6RSS PM without HKPs is built first, thereafter, the two HKP constraints are modelled, to achieve the model of the RAPM of interest.

### 5.1. Model 1

At first, the mechanism is virtually cut at *M_i_*, then the dynamics of the end effector and the chains are formulated in the task space and the joint space independently. Next, in view of the closed-loop constraints in reality, the constraint forces using the Lagrange multipliers are added to generate the complete dynamics model of the RAPM. Finally, these multipliers are eliminated by virtue of the null-space method, reaching the dynamics model sought.

#### 5.1.1. The End Effector of the RAPM

In view of the translational and the angular velocities of the end effector in Equations (8) and (10), respectively, its kinetic energy is

(39) $$T_{EE1}=\frac{1}{2}\cdot {\left[\begin{array}{c} V_{O_{M}}\\ \omega_{EE} \end{array}\right]}^{T}\cdot \left[\begin{array}{cc} m_{EE}\cdot E_{3} & \\ & I_{EE} \end{array}\right]\cdot \left[\begin{array}{c} V_{O_{M}}\\ \omega_{EE} \end{array}\right]=\frac{1}{2}\cdot {\dot{q}}_{EE}^{T}\cdot M_{EE1}\cdot {\dot{q}}_{EE}$$

where $M_{EE1}$ is its 4×4 mass matrix that can be expressed in a closed-form as

(40) $$M_{EE1}={\left[\begin{array}{c} M_{1b}\\ M_{0b} \end{array}\right]}^{T}\cdot \left[\begin{array}{cc} m_{EE}\cdot E_{3} & \\ & I_{EE} \end{array}\right]\cdot \left[\begin{array}{c} M_{1b}\\ M_{0b} \end{array}\right]$$

Its potential energy is

(41) $$P_{EE1}=m_{EE}\cdot g\cdot Z$$

Regarding these, its Lagrange function is

(42) $$L_{EE1}=T_{EE1}-P_{EE1}=\frac{1}{2}\cdot {\dot{q}}_{EE}^{T}\cdot M_{EE1}\cdot {\dot{q}}_{EE}-m_{EE}\cdot g\cdot Z$$

One can derive that

(43) $$\begin{array}{l} \frac{\partial L_{EE1}}{\partial {\dot{q}}_{EE}}=M_{EE1}\left(q_{EE}\right)\cdot {\dot{q}}_{EE}\\ \frac{d}{dt}\left(\frac{\partial L_{EE1}}{\partial {\dot{q}}_{EE}}\right)=M_{EE1}\left(q_{EE}\right)\cdot {\ddot{q}}_{EE}+\frac{\partial M_{EE1}\left(q_{EE}\right)}{\partial q_{EE}^{T}}\cdot \left({\dot{q}}_{EE}⊗E_{4}\right)\cdot {\dot{q}}_{EE}\\ \frac{\partial L_{EE1}}{\partial q_{EE}}=\frac{1}{2}{\left(E_{4}⊗{\dot{q}}_{EE}\right)}^{T}\cdot \frac{\partial M_{EE1}\left(q_{EE}\right)}{\partial q_{EE}}\cdot {\dot{q}}_{EE}-m_{EE}\cdot g\cdot \frac{\partial Z}{\partial q_{EE}} \end{array}$$

by virtue of which the Lagrangian equation of the end effector is

(44) $$\frac{d}{dt}\left(\frac{\partial L_{EE1}}{\partial {\dot{q}}_{EE}}\right)-\frac{\partial L_{EE1}}{\partial q_{EE}}=M_{EE1}\left(q_{EE}\right)\cdot {\ddot{q}}_{EE}+C_{EE1}\left(q_{EE},{\dot{q}}_{EE}\right)\cdot {\dot{q}}_{EE}+G_{EE1}\left(q_{EE}\right)=F_{EE1}$$

where

$$\begin{array}{l} C_{EE1}\left(q_{EE},{\dot{q}}_{EE}\right)=\frac{\partial M_{EE1}\left(q_{EE}\right)}{\partial q_{EE}^{T}}\cdot \left({\dot{q}}_{EE}⊗E_{4}\right)-\frac{1}{2}{\left(E_{4}⊗{\dot{q}}_{EE}\right)}^{T}\cdot \frac{\partial M_{EE1}\left(q_{EE}\right)}{\partial q_{EE}}\\ G_{EE1}\left(q_{EE}\right)=m_{EE}\cdot g\cdot \frac{\partial Z}{\partial q_{EE}} \end{array}$$

are the 4×4 Coriolis and centrifugal force matrix, and the 4×1 gravitational force vector of the end effector, respectively. $F_{EE1}$ is the 4×1 generalised force vector corresponding to the four DOFs of the end effector. Because the 3×1 bite force ***F****_B_* is measured in {*S*}, it must be transferred into the directions of the four DOFs as follows:

The instantaneous power exerted by ***F****_B_* is

(45) $${\left(\left[\begin{array}{c} E_{3}\\ O_{M}B\times \end{array}\right]\cdot F_{B}\right)}^{T}\cdot \left[\begin{array}{c} V_{O_{M}}\\ \omega_{EE} \end{array}\right]=F_{B}^{T}\cdot \begin{array}{cc} {}[E_{3} & {\left(O_{M}B\times \right)}^{T}] \end{array}\cdot \left[\begin{array}{c} M_{1b}\\ M_{0b} \end{array}\right]\cdot {\dot{q}}_{EE}=F_{EE1}^{T}\cdot {\dot{q}}_{EE}$$

Hence, the generalized force vector can be mapped by ***F****_B_* as

(46) $$F_{EE1}={\left([E_{3}{\left(O_{M}B\times \right)}^{T}]\cdot \left[\begin{array}{c} M_{1b}\\ M_{0b} \end{array}\right]\right)}^{T}\cdot F_{B}$$

Now the dynamics model of the end effector has been formulated straightforwardly in the task space.

#### 5.1.2. The *i*th Chain

For the coupler ***S****_i_****M****_i_*, its kinetic energy can be computed as

(47) $$T_{S_{i}M_{i}}=\frac{1}{2}\cdot {\left[\begin{array}{c} V_{E_{i}}\\ \omega_{S_{i}M_{i}} \end{array}\right]}^{T}\cdot \left[\begin{array}{cc} m_{S_{i}M_{i}}\cdot E_{3} & \\ & I_{S_{i}M_{i}} \end{array}\right]\cdot \left[\begin{array}{c} V_{E_{i}}\\ \omega_{S_{i}M_{i}} \end{array}\right]$$

Upon substitution of Equations (20) and (23) into Equation (47), it produces

(48) $$T_{S_{i}M_{i}}=\frac{1}{2}\cdot {\dot{q}}_{r_{i}}^{T}\cdot M_{S_{i}M_{i}}\cdot {\dot{q}}_{r_{i}}$$

where $M_{S_{i}M_{i}}$ is the mass matrix of ***S****_i_****M****_i_* and can be expressed as

MSiMi=JEiRNi0S⋅RωSiMiT⋅mSiMi⋅E3ISiMi⋅JEiRNi0S⋅RωSiMi

The potential energy of ***S****_i_****M****_i_* is

(49) $$P_{S_{i}M_{i}}=m_{S_{i}M_{i}}\cdot g\cdot O_{S}E_{i\left(3\right)}$$

where $O_{S}E_{i\left(3\right)}$ is the third term of $O_{S}E_{i}$.

The crank ***G****_i_****S****_i_* only rotates around the $Z_{C_{i}}$ axis of $\left\{C_{i}\right\}$, thus its kinetic energy is

(50) $$T_{G_{i}S_{i}}=\frac{1}{2}I_{G_{i}S_{i}}{\dot{\theta }}_{i}^{2}=\frac{1}{2}{\dot{q}}_{r_{i}}^{T}\cdot M_{G_{i}S_{i}}\cdot {\dot{q}}_{r_{i}}$$

where $M_{G_{i}S_{i}}={\left[\begin{array}{cc} 1 & 0_{1\times 2} \end{array}\right]}^{T}\cdot I_{G_{i}S_{i}}\cdot \left[\begin{array}{cc} 1 & 0_{1\times 2} \end{array}\right]$ is its 3×3 mass matrix. The mass centre of the crank lies on the $Z_{C_{i}}$ axis, thus its potential energy $P_{G_{i}S_{i}}$ is a constant. Regarding these, the kinetic energy, the potential energy, and the Lagrangian function of the *i*th chain are computed sequentially as

(51) $$\begin{array}{l} T_{G_{i}S_{i}M_{i}}\left(q_{r_{i}},{\dot{q}}_{r_{i}}\right)=T_{S_{i}M_{i}}+T_{G_{i}S_{i}}=\frac{1}{2}{\dot{q}}_{r_{i}}^{T}\cdot M_{G_{i}S_{i}M_{i}}\left(q_{r_{i}}\right)\cdot {\dot{q}}_{r_{i}}\\ P_{G_{i}S_{i}M_{i}}\left(q_{r_{i}}\right)=P_{S_{i}M_{i}}+P_{G_{i}S_{i}}\\ L_{i}=T_{G_{i}S_{i}M_{i}}\left(q_{r_{i}},{\dot{q}}_{r_{i}}\right)-P_{G_{i}S_{i}M_{i}}\left(q_{r_{i}}\right) \end{array}$$

As such, one can compute that

(52) $$\begin{array}{c} \frac{\partial L_{i}}{\partial {\dot{q}}_{r_{i}}}=M_{G_{i}S_{i}M_{i}}\left(q_{r_{i}}\right)\cdot {\dot{q}}_{r_{i}}\\ \frac{d}{dt}\left(\frac{\partial L_{i}}{\partial {\dot{q}}_{r_{i}}}\right)=M_{G_{i}S_{i}M_{i}}\left(q_{r_{i}}\right)\cdot {\ddot{q}}_{r_{i}}+\frac{\partial M_{G_{i}S_{i}M_{i}}}{\partial q_{r_{i}}^{T}}\cdot \left({\dot{q}}_{r_{i}}⊗E_{3}\right)\cdot {\dot{q}}_{r_{i}}\\ \frac{\partial L_{i}}{\partial q_{r_{i}}}=\frac{1}{2}\cdot {\left(E_{3}⊗{\dot{q}}_{r_{i}}\right)}^{T}\cdot \frac{\partial M_{G_{i}S_{i}M_{i}}}{\partial q_{r_{i}}}\cdot {\dot{q}}_{r_{i}}-m_{S_{i}M_{i}}\cdot g\cdot \frac{\partial O_{S}E_{i\left(3\right)}}{\partial q_{r_{i}}} \end{array}$$

The Lagrangian formulation of the *i*th chain is

(53) $$M_{G_{i}S_{i}M_{i}}\left(q_{r_{i}}\right)\cdot {\ddot{q}}_{r_{i}}+C_{G_{i}S_{i}M_{i}}\left(q_{r_{i}},{\dot{q}}_{r_{i}}\right)\cdot {\dot{q}}_{r_{i}}+G_{G_{i}S_{i}M_{i}}\left(q_{r_{i}}\right)=F_{G_{i}S_{i}M_{i}}\left(\tau_{i}\right)$$

where $M_{G_{i}S_{i}M_{i}}\left(q_{r_{i}}\right),C_{G_{i}S_{i}M_{i}}\left(q_{r_{i}},{\dot{q}}_{r_{i}}\right),G_{G_{i}S_{i}M_{i}}\left(q_{r_{i}}\right)$ and $F_{G_{i}S_{i}M_{i}}\left(\tau_{i}\right)$ are the 3×3 mass matrix, the 3×3 Coriolis and centrifugal force matrix, the 3×1 gravitational force vector, and the 3×1 generalized force vector, respectively. They can be expressed sequentially as

(54) $$\begin{array}{l} M_{G_{i}S_{i}M_{i}}\left(q_{r_{i}}\right)=M_{S_{i}M_{i}}+M_{G_{i}S_{i}}\\ C_{G_{i}S_{i}M_{i}}\left(q_{r_{i}},{\dot{q}}_{r_{i}}\right)=\frac{\partial M_{G_{i}S_{i}M_{i}}}{\partial q_{r_{i}}^{T}}\cdot \left({\dot{q}}_{r_{i}}⊗E_{3}\right)-\frac{1}{2}\cdot {\left(E_{3}⊗{\dot{q}}_{r_{i}}\right)}^{T}\cdot \frac{\partial M_{G_{i}S_{i}M_{i}}}{\partial q_{r_{i}}}\\ G_{G_{i}S_{i}M_{i}}\left(q_{r_{i}}\right)=m_{S_{i}M_{i}}\cdot g\cdot \frac{\partial O_{S}E_{i\left(3\right)}}{\partial q_{r_{i}}}\\ F_{G_{i}S_{i}M_{i}}=\left[\begin{array}{c} \tau_{i}\\ 0_{2\times 1} \end{array}\right] \end{array}$$

Now the model of the *i*th chain has been conveniently built in its joint space.

#### 5.1.3. The Entire Mechanism

For the complete mechanism, it can be formatted that

(55) $$M_{1}\left(q_{1}\right)\cdot {\ddot{q}}_{1}+C_{1}\left(q_{1},{\dot{q}}_{1}\right)\cdot {\dot{q}}_{1}+G_{1}\left(q_{1}\right)=F_{1}\left(\tau ,q_{EE}\right)$$

where

$$\begin{array}{l} q_{1}={\left[\begin{array}{c} q_{r_{1}}\left(q_{EE}\right)\\ \vdots \\ q_{r_{6}}\left(q_{EE}\right)\\ q_{EE} \end{array}\right]}_{22\times 1}\;,\;M_{1}\left(q_{1}\right)={\left[\begin{array}{cccc} M_{G_{1}S_{1}M_{1}}\left(q_{r_{1}}\right) & & & \\ & \ddots & & \\ & & M_{G_{6}S_{6}M_{6}}\left(q_{r_{6}}\right) & \\ & & & M_{EE1}\left(q_{EE}\right) \end{array}\right]}_{22\times 22}\;,\\ C_{1}\left(q_{1},{\dot{q}}_{1}\right)={\left[\begin{array}{cccc} C_{G_{1}S_{1}M_{1}}\left(q_{r_{1}},{\dot{q}}_{r_{1}}\right) & & & \\ & \ddots & & \\ & & C_{G_{6}S_{6}M_{6}}\left(q_{r_{6}},{\dot{q}}_{r_{6}}\right) & \\ & & & C_{EE1}\left(q_{EE},{\dot{q}}_{EE}\right) \end{array}\right]}_{22\times 22},\;G_{1}\left(q_{1}\right)={\left[\begin{array}{c} G_{G_{1}S_{1}M_{1}}\left(q_{r_{1}}\right)\\ \vdots \\ G_{G_{6}S_{6}M_{6}}\left(q_{r_{6}}\right)\\ G_{EE1}\left(q_{EE}\right) \end{array}\right]}_{22\times 1},\;F_{1}\left(\tau ,q_{EE}\right)={\left[\begin{array}{c} F_{G_{1}S_{1}M_{1}}\left(\tau_{1}\right)\\ \vdots \\ F_{G_{6}S_{6}M_{6}}\left(\tau_{6}\right)\\ F_{EE1}\left(q_{EE}\right) \end{array}\right]}_{22\times 1} \end{array}$$

are the generalised coordinate vector, the mass matrix, the Coriolis and centrifugal force matrix, the gravitational force vector, and the generalised external force vector free of closed-loop constraints, respectively. Their sizes are given in the subscripts on the right. Physically, there are 18 holonomic constraint equations in total from the closed loops by the kinematic chains and the end effector, and they are determined as

(56) $$\Phi =\left[\begin{array}{c} O_{S}G_{1}+G_{1}S_{1}+S_{1}M_{1}-O_{S}O_{M}-O_{M}M_{1}=0_{3\times 1}\\ \vdots \\ O_{S}G_{6}+G_{6}S_{6}+S_{6}M_{6}-O_{S}O_{M}-O_{M}M_{6}=0_{3\times 1} \end{array}\right]$$

The complete dynamics model is built as

(57) $$M_{1}\left(q_{1}\right)\cdot {\ddot{q}}_{1}+C_{1}\left(q_{1},{\dot{q}}_{1}\right)\cdot {\dot{q}}_{1}+G_{1}\left(q_{1}\right)+\Phi_{q_{1}}^{T}\cdot \lambda_{1}=F_{1}\left(\tau ,q_{EE}\right)$$

where $\Phi_{q_{1}}=Jacobian\left(\begin{array}{cc} \Phi , & q_{1} \end{array}\right)$ is the 18×22 constraint Jacobian matrix, $\lambda_{1}$ is the 18×1 unknown Lagrangian multiplier vector, which means the magnitudes of the generalized constraint forces $\Phi_{q_{1}}^{T}\cdot \lambda_{1}$ in the mechanism at hand.

From Section 3.2, the 22 terms in $q_{1}$ are not all independent: $q_{r_{i}}\left(i=1,\ldots ,6\right)$ is the function of $q_{EE}$ whose four elements are independent, thus $q_{1}$ cannot serve as an independent generalised coordinate vector. In the meantime, from Equations (17) and (19), one can find that

(58) $$\begin{array}{l} {\dot{q}}_{1}=M_{p1}\cdot {\dot{q}}_{EE}\\ {\ddot{q}}_{1}=M_{p1}\cdot {\ddot{q}}_{EE}+M_{p2}\cdot {\dot{q}}_{EE} \end{array}$$

where

$$M_{p1}={\left[\begin{array}{c} M_{31}\\ \vdots \\ M_{36}\\ E_{4} \end{array}\right]}_{22\times 4},M_{p2}={\left[\begin{array}{c} M_{41}\\ \vdots \\ M_{46}\\ 0_{4} \end{array}\right]}_{22\times 4}$$

Substituting Equation (58) into Equation (57) yields

(59) $$M_{1}\left(q_{1}\right)\cdot \left(M_{p1}\cdot {\ddot{q}}_{EE}+M_{p2}\cdot {\dot{q}}_{EE}\right)+C_{1}\left(q_{1},{\dot{q}}_{1}\right)\cdot M_{p1}\cdot {\dot{q}}_{EE}+G\left(q_{1}\right)+\Phi_{q_{1}}^{T}\cdot \lambda_{1}=F_{1}\left(\tau ,q_{EE}\right)$$

where $q_{1}$ and ${\dot{q}}_{1}$ in $M_{1}\left(q_{1}\right)$, $C_{1}\left(q_{1},{\dot{q}}_{1}\right)$ and $G_{1}\left(q_{1}\right)$ can be numerically computed by $q_{EE}$ and ${\dot{q}}_{EE}$. To avoid computing $\lambda_{1}$, the null space method is used: differentiating the constraint equations in Equation (56) with respect to time gives rise to

(60) $$\dot{\Phi }=\Phi_{q_{1}}\cdot {\dot{q}}_{1}=0_{18\times 1}$$

Upon substitution of the first equation in Equation (58) into Equation (60), it results in

(61) $$\dot{\Phi }=\Phi_{q_{1}}\cdot M_{p1}\cdot {\dot{q}}_{EE}=0_{18\times 1}$$

In it, ${\dot{q}}_{EE}$ is the first time derivative of the independent generalised coordinate vector $q_{EE}$ and it can be assigned arbitrarily. However, while doing so, it must hold that

(62) $$\Phi_{q_{1}}\cdot M_{p1}=0_{18\times 4}$$

or equivalently, $M_{p1}^{T}\cdot \Phi_{q_{1}}^{T}=0_{4\times 18}$. Thereby, multiplying both sides of Equation (59) by $M_{p1}^{T}$ from the left gives rise to

(63) $$M_{p1}^{T}\cdot M_{1}\left(q_{1}\right)\cdot \left(M_{p1}\cdot {\ddot{q}}_{EE}+M_{p2}\cdot {\dot{q}}_{EE}\right)+M_{p1}^{T}\cdot C_{1}\left(q_{1},{\dot{q}}_{1}\right)\cdot M_{p1}\cdot {\dot{q}}_{EE}+M_{p1}^{T}\cdot G_{1}\left(q_{1}\right)=M_{p1}^{T}\cdot F_{1}\left(\tau ,q_{EE}\right)$$

In this manner, it is not necessary to compute $\lambda_{1}$. This equation can be rewritten as

(64) $$M_{r1}\cdot {\ddot{q}}_{EE}+C_{r1}\cdot {\dot{q}}_{EE}+G_{r1}=F_{r1}=J_{\theta 1}^{T}\cdot \tau +F_{EE1}$$

where

$$\begin{array}{l} M_{r1}=M_{p1}^{T}\cdot M_{1}\left(q_{1}\right)\cdot M_{p1}\\ C_{r1}=M_{p1}^{T}\cdot \left(M_{1}\left(q_{1}\right)\cdot M_{p2}+C_{1}\left(q_{1},{\dot{q}}_{1}\right)\cdot M_{p1}\right)\\ G_{r1}=M_{p1}^{T}\cdot G_{1}\left(q_{1}\right)\\ J_{\theta 1}=\left[\begin{array}{c} M_{31\left(1,:\right)}\\ \vdots \\ M_{36\left(1,:\right)} \end{array}\right] \end{array}$$

and $J_{\theta 1}$ actually denotes the 6×4 Jacobian matrix between θ and $q_{EE}$, i.e.,

(65) $$\begin{array}{l} J_{\theta 1}=\mathrm{Jacobian}\left(\begin{array}{cc} \theta , & q_{EE} \end{array}\right)\\ \dot{\theta }=J_{\theta 1}\cdot {\dot{q}}_{EE} \end{array}$$

In Equation (64), there are four equations and six unknowns, indicating the 4-DOF mechanism is actuated by six actuators.

### 5.2. Model 2

It is recalled that the conversion of two DOFs *Z* and γ into parasitic motions in the RAPM is caused by the two HKP constraints onto the end effector as in Section 3.1. An alternative though the intuitive and simple approach is presented in this section: the idea is to first model the inverse dynamics of the 6RSS PM, then on this basis, the HKP constraints are modelled to arrive at the solution of the RAPM at hand.

#### 5.2.1. The End Effector of the 6RSS PM

In the 6RSS PM, the six DOFs of the end effector can be expressed in Equation (3), and its angular velocity, the translational velocity of the mass centre ***O****_M_* can be computed as

(66) $$\begin{array}{l} \omega_{EE}=M_{0a}\cdot {\dot{X}}_{EE}\\ V_{O_{M}}=M_{1a}\cdot {\dot{X}}_{EE} \end{array}$$

where $M_{0a}$ and $M_{1a}$ are recalled in Equations (8) and (10), respectively. Its kinetic energy is

(67) $$T_{EE2}=\frac{1}{2}\cdot {\dot{X}}_{EE}^{T}\cdot M_{EE}\cdot {\dot{X}}_{EE}$$

where $M_{EE2}={\left[\begin{array}{c} M_{1a}\\ M_{0a} \end{array}\right]}^{T}\cdot \left[\begin{array}{cc} m_{EE}\cdot E_{3} & \\ & I_{EE} \end{array}\right]\cdot \left[\begin{array}{c} M_{1a}\\ M_{0a} \end{array}\right]$ denotes its 6×6 mass matrix. Its potential energy is

(68) $$P_{EE1}=m_{EE}\cdot g\cdot Z$$

It is remarked that though the expressions of Equations (41) and (68) are exactly identical, *Z* in Equation (41) is the function of ***q****_EE_* in the RAPM, while in Equation (68), *Z* is a DOF of the 6RSS PM. The Lagrange function is

(69) $$L_{EE2}=T_{EE2}-P_{EE2}=\frac{1}{2}\cdot {\dot{X}}_{EE}^{T}\cdot M_{EE2}\cdot {\dot{X}}_{EE}-m_{EE}\cdot g\cdot Z$$

By virtue of it, one can obtain that

(70) $$\begin{array}{l} \frac{\partial L_{EE2}}{\partial {\dot{X}}_{EE}}=M_{EE2}\left(X_{EE}\right)\cdot {\dot{X}}_{EE}\\ \frac{d}{dt}\left(\frac{\partial L_{EE2}}{\partial {\dot{X}}_{EE}}\right)=M_{EE2}\left(X_{EE}\right)\cdot {\ddot{X}}_{EE}+\frac{\partial M_{EE2}\left(X_{EE}\right)}{\partial X_{EE}^{T}}\cdot \left({\dot{X}}_{EE}⊗E_{6}\right)\cdot {\dot{X}}_{EE}\\ \frac{\partial L_{EE2}}{\partial X_{EE}}=\frac{1}{2}{\left(E_{6}⊗{\dot{X}}_{EE}\right)}^{T}\cdot \frac{\partial M_{EE2}\left(X_{EE}\right)}{\partial X_{EE}}\cdot {\dot{X}}_{EE}-m_{EE}\cdot g\cdot \frac{\partial Z}{\partial X_{EE}} \end{array}$$

The instantaneous power exerted by the bite force ***F****_B_* is

(71) $$F_{B}^{T}\cdot V_{O_{M}}+{\left(O_{M}B\times F_{B}\right)}^{T}\cdot \omega_{EE}=F_{EE2}^{T}\cdot {\dot{X}}_{EE}$$

where $F_{EE2}$ is the generalised force vector corresponding to $X_{EE}$ and it is computed as

(72) $$F_{EE2}=M_{2a}^{T}\cdot F_{B}$$

where $M_{2a}=\left[\begin{array}{cc} E_{3} & {\left(O_{M}B\times \right)}^{T} \end{array}\right]\cdot \left[\begin{array}{c} M_{1a}\\ M_{0a} \end{array}\right]$. From Equations (70) and (72), the dynamics model of the end effector can be written as

(73) $$M_{EE2}\left(X_{EE}\right)\cdot {\ddot{X}}_{EE}+C_{EE2}\left(X_{EE},{\dot{X}}_{EE}\right)\cdot {\dot{X}}_{EE}+G_{EE2}\left(X_{EE}\right)=M_{2a}^{T}\cdot F_{B}$$

where

$$\begin{array}{l} C_{EE2}\left(X_{EE},{\dot{X}}_{EE}\right)=\frac{\partial M_{EE2}\left(X_{EE}\right)}{\partial X_{EE}^{T}}\cdot \left({\dot{X}}_{EE}⊗E_{6}\right)-\frac{1}{2}{\left(E_{6}⊗{\dot{X}}_{EE}\right)}^{T}\cdot \frac{\partial M_{EE2}\left(X_{EE}\right)}{\partial X_{EE}}\\ G_{EE2}\left(q_{EE}\right)=m_{EE}\cdot g\cdot \frac{\partial Z}{\partial X_{EE}} \end{array}$$

are the 6×6 Coriolis and centrifugal force matrix and the 6×1 gravitational force vector of the end effector, respectively.

#### 5.2.2. The Entire Mechanism

The dynamics model of the *i*th (*i* = 1, …, 6) chain is computed identically as that in Section 5.1.2, but it must be remembered that now $q_{r_{i}},{\dot{q}}_{r_{i}},{\ddot{q}}_{r_{i}}$ are functions of $X_{EE},{\dot{X}}_{EE},{\ddot{X}}_{EE}$, i.e.,

$$\begin{array}{l} q_{r_{i}}=q_{r_{i}}\left(X_{EE}\right)\\ {\dot{q}}_{r_{i}}={\dot{q}}_{r_{i}}\left(X_{EE},{\dot{X}}_{EE}\right)\\ {\ddot{q}}_{r_{i}}={\ddot{q}}_{r_{i}}\left(X_{EE},{\dot{X}}_{EE},{\ddot{X}}_{EE}\right) \end{array}$$

Differentiating Equation (15) with respect to time again results in

(74) $$M_{1i}\cdot {\dot{q}}_{r_{i}}={\bar{M}}_{2i}\cdot {\dot{X}}_{EE}$$

where ${\bar{M}}_{2i}=\mathrm{Jacobian}\begin{array}{cc} (O_{S}O_{M}+O_{M}M_{i}, & X_{EE}) \end{array}$ and its size is 3×6. Thereby, it can be further found that

(75) $$\begin{array}{l} {\dot{q}}_{r_{i}}={\bar{M}}_{3i}\cdot {\dot{X}}_{EE}\\ {\ddot{q}}_{r_{i}}={\bar{M}}_{3i}\cdot {\ddot{X}}_{EE}+{\bar{M}}_{4i}\cdot {\dot{X}}_{EE} \end{array}$$

where

$$\begin{array}{l} {\bar{M}}_{3i}=M_{1i}^{-1}\cdot {\bar{M}}_{2i}\\ M_{4i}=M_{1i}^{-1}\cdot \left({\dot{\bar{M}}}_{2i}-{\dot{M}}_{1i}\cdot {\bar{M}}_{3i}\right)\\ {\dot{\bar{M}}}_{2i}=\frac{\partial {\bar{M}}_{2i}}{\partial X_{EE}^{T}}\cdot \left({\dot{X}}_{EE}⊗E_{6}\right) \end{array}$$

The dynamics model of the mechanism free of closed-loop constraints can be built as

(76) $$M_{2}\left(q_{2}\right)\cdot {\ddot{q}}_{2}+C_{2}\left(q_{2},{\dot{q}}_{2}\right)\cdot {\dot{q}}_{2}+G_{2}\left(q_{2}\right)=F_{2}$$

where

$$\begin{array}{l} q_{2}={\left[\begin{array}{c} q_{r_{1}}\left(X_{EE}\right)\\ \vdots \\ q_{r_{6}}\left(X_{EE}\right)\\ X_{EE} \end{array}\right]}_{24\times 1},\;M_{2}\left(q_{2}\right)={\left[\begin{array}{cccc} M_{G_{1}S_{1}M_{1}}\left(q_{r_{1}}\right) & & & \\ & \ddots & & \\ & & M_{G_{6}S_{6}M_{6}}\left(q_{r_{6}}\right) & \\ & & & M_{EE2}\left(X_{EE}\right) \end{array}\right]}_{24\times 24}\;,\\ C_{2}\left(q_{2},{\dot{q}}_{2}\right)={\left[\begin{array}{cccc} C_{G_{1}S_{1}M_{1}}\left(q_{r_{1}},{\dot{q}}_{r_{1}}\right) & & & \\ & \ddots & & \\ & & C_{G_{6}S_{6}M_{6}}\left(q_{r_{6}},{\dot{q}}_{r_{6}}\right) & \\ & & & C_{EE2}\left(X_{EE},{\dot{X}}_{EE}\right) \end{array}\right]}_{24\times 24},\;G_{2}\left(q_{2}\right)={\left[\begin{array}{c} G_{G_{1}S_{1}M_{1}}\left(q_{r_{1}}\right)\\ \vdots \\ G_{G_{6}S_{6}M_{6}}\left(q_{r_{6}}\right)\\ G_{EE2}\left(X_{EE}\right) \end{array}\right]}_{24\times 1},\;F_{2}\left(q_{2}\right)={\left[\begin{array}{c} F_{G_{1}S_{1}M_{1}}\left(\tau_{1}\right)\\ \vdots \\ F_{G_{6}S_{6}M_{6}}\left(\tau_{6}\right)\\ F_{EE2}\left(X_{EE}\right) \end{array}\right]}_{24\times 1} \end{array}$$

The constraint equation vector is as that in Equation (56), and the complete dynamics model of the mechanism with closed-loop constraints is

(77) $$M_{2}\left(q_{2}\right){\ddot{q}}_{2}+C_{2}\left(q_{2},{\dot{q}}_{2}\right)\cdot {\dot{q}}_{2}+G_{2}\left(q_{2}\right)+\Phi_{q_{2}}^{T}\cdot \lambda_{2}=F_{2}\left(\tau ,X_{EE}\right)$$

where $\Phi_{q_{2}}=\mathrm{Jacobian}\left(\begin{array}{cc} \Phi , & q_{2} \end{array}\right)$ is the 18×24 constraint Jacobian matrix, $\lambda_{2}$ is the 18×1 unknown Lagrangian multiplier vector which means the magnitudes of the generalised constraint forces $\Phi_{q2}^{T}\cdot \lambda_{2}$ in the 6RSS PM.

By virtue of the identical manner as in Section 5.1.3, one can derive that

(78) $$\begin{array}{l} {\dot{q}}_{2}={\bar{M}}_{p1}\cdot {\dot{X}}_{EE}\\ {\ddot{q}}_{2}={\bar{M}}_{p1}\cdot {\ddot{X}}_{EE}+{\bar{M}}_{p2}\cdot {\dot{X}}_{EE} \end{array}$$

where ${\bar{M}}_{p1}={\left[\begin{array}{c} {\bar{M}}_{31}\\ \vdots \\ {\bar{M}}_{36}\\ E_{6} \end{array}\right]}_{24\times 6},{\bar{M}}_{p2}={\left[\begin{array}{c} {\bar{M}}_{41}\\ \vdots \\ {\bar{M}}_{46}\\ 0_{6} \end{array}\right]}_{24\times 6}$. Upon substitution of Equation (78) into Equation (77), it gives rise to

(79) $$M_{2}\left(q_{2}\right)\cdot \left({\bar{M}}_{p1}\cdot {\ddot{X}}_{EE}+{\bar{M}}_{p2}\cdot {\dot{X}}_{EE}\right)+C_{2}\left(q_{2},{\dot{q}}_{2}\right)\cdot {\bar{M}}_{p1}\cdot {\dot{X}}_{EE}+G_{2}\left(q_{2}\right)+\Phi_{q2}^{T}\cdot \lambda_{2}=F_{2}\left(\tau ,X_{EE}\right)$$

Identically, using the null space method as in Section 5.1.3, the Lagrangian multiplier vector $\lambda_{2}$ is eliminated by multiplying Equation (79) with ${\bar{M}}_{p1}^{T}$, and it produces that

(80) $$M_{r2}\cdot {\ddot{X}}_{EE}+C_{r2}\cdot {\dot{X}}_{EE}+G_{r2}=M_{p1}^{T}\cdot F_{2}=J_{\theta 2}^{T}\cdot \tau +F_{EE2}$$

where

$$\begin{array}{l} M_{r2}={\bar{M}}_{p1}^{T}\cdot M_{2}\left(q_{2}\right)\cdot {\bar{M}}_{p1}\\ C_{r2}={\bar{M}}_{p1}^{T}\cdot \left(M_{2}\left(q_{2}\right)\cdot {\bar{M}}_{p2}+C_{2}\left(q_{2},{\dot{q}}_{2}\right)\cdot {\bar{M}}_{p1}\right)\\ G_{r2}={\bar{M}}_{p1}^{T}\cdot G_{2}\left(q_{2}\right)\\ J_{\theta 2}={\left[\begin{array}{c} {\bar{M}}_{31\left(1,:\right)}\\ \vdots \\ {\bar{M}}_{36\left(1,:\right)} \end{array}\right]}_{6\times 6} \end{array}$$

and $J_{\theta 2}$ actually denotes the 6×6 Jacobian matrix between θ and $X_{EE}$, i.e.,

(81) $$\begin{array}{l} J_{\theta 2}=\mathrm{Jacobian}\left(\begin{array}{cc} \theta , & X_{EE} \end{array}\right)\\ \dot{\theta }=J_{\theta 2}\cdot {\dot{X}}_{EE} \end{array}$$

In Equation (80), there are six equations and six unknowns. By adding the two HKP constraints, the DOFs have been transferred from $X_{EE}$ to $q_{EE}$, thereby, using Equation (9), one can derive that

(82) $${\ddot{X}}_{EE}=M_{J}\cdot {\ddot{q}}_{EE}+{\dot{M}}_{J}\cdot {\dot{q}}_{EE}$$

where ${\dot{M}}_{J}=\frac{\partial M_{J}}{\partial q_{EE}^{T}}\cdot \left({\dot{q}}_{EE}⊗E_{4}\right)$. Substituting Equations (9) and (82) into Equation (80) generates

(83) $$M_{r2}\cdot M_{J}\cdot {\ddot{q}}_{EE}+\left(M_{r2}\cdot {\dot{M}}_{J}+C_{r2}\cdot M_{J}\right)\cdot {\dot{q}}_{EE}+G_{r2}-F_{EE2}=J_{\theta 2}^{T}\cdot \tau$$

Multiplying both sides of Equation (83) with $M_{J}^{T}$ yields

(84a) $$M_{J}^{T}\cdot M_{r2}\cdot M_{J}\cdot {\ddot{q}}_{EE}+M_{J}^{T}\cdot \left(M_{r2}\cdot {\dot{M}}_{J}+C_{r2}\cdot M_{J}\right)\cdot {\dot{q}}_{EE}+M_{J}^{T}\cdot \left(G_{r2}-F_{EE2}\right)={\left(J_{\theta 2}\cdot M_{J}\right)}^{T}\cdot \tau$$

in which the 4×4 generalised mass matrix $M_{J}^{T}\cdot M_{r2}\cdot M_{J}$ is square. The size of $J_{\theta 2}\cdot M_{J}$ is 6×4, thus there are six unknowns and four equations. It is worth mentioning that all the matrices and vectors in Equation (84a) are ultimately functions of $q_{EE},{\dot{q}}_{EE},{\ddot{q}}_{EE}$, rather than $X_{EE},{\dot{X}}_{EE},{\ddot{X}}_{EE}$.

Or, equivalently and more conveniently, $M_{J}^{T}$ can be directly multiplied to the two sides of Equation (80)

(84b) $$M_{J}^{T}\cdot \left(M_{r2}\cdot {\ddot{X}}_{EE}+C_{r2}\cdot {\dot{X}}_{EE}+G_{r2}-F_{EE2}\right)={\left(J_{\theta 2}\cdot M_{J}\right)}^{T}\cdot \tau$$

**Figure 6:**
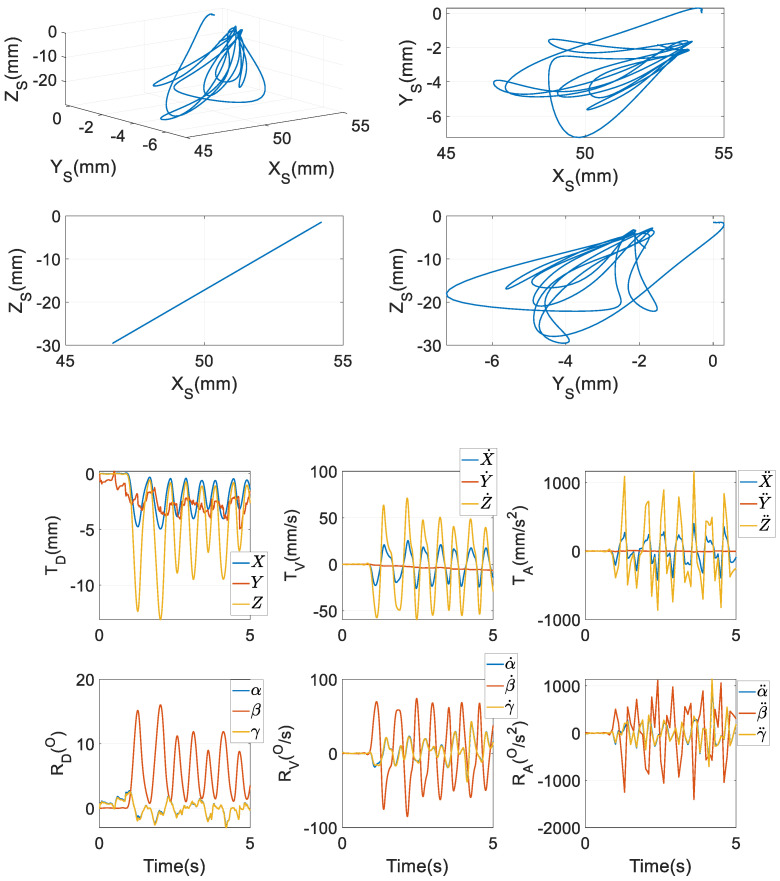
Motions of the end effector in 3D space and time history.

It is known that $X_{EE},{\dot{X}}_{EE},{\ddot{X}}_{EE}$ are functions of $q_{EE},{\dot{q}}_{EE},{\ddot{q}}_{EE}$, and the numerical values of the former can be computed by those of the latter, as obtained and will be shown in the last six subplots in Figure 6. In this manner, all the matrices and vectors in the bracket of the left-hand side of Equation (84b) can be directly computed by $X_{EE},{\dot{X}}_{EE},{\ddot{X}}_{EE}$ rather than $q_{EE},{\dot{q}}_{EE},{\ddot{q}}_{EE}$, in spite of the fact that they are ultimately functions of $q_{EE},{\dot{q}}_{EE},{\ddot{q}}_{EE}$. In other words, the dynamics model of the RAPM can be conveniently generated by formatting the model of the 6RSS PM as its core firstly; next in modelling the two HKP constraints, only the numerical multiplication of $M_{J}^{T}$ is needed. In the practical application of Model 2, Equation (84b) would be directly used rather than Equation (84a). It will be shown in Section 7.4.3 how improvements in computational efficiency can be affected by using the dynamics model of the 6RSS PM as the core as exhibited in Equation (84b) to formulate explicit equations of motion.

## 6. Principle of Virtual Work

### 6.1. Model 1

In using the principle of virtual work, it also must be emphasised that $F_{T_{L}}$ and $F_{T_{R}}$ at the two HKPs are ideal constraint forces that are not involved with virtual work, thus they must not appear in the model. Thereby, the 6×1 resultants of applied and inertia wrenches exerted at the centre of the end effector are

(85) $$W_{EE}=-M_{3b}$$

The one-dimensional resultant torque acting at the crank ***G****_i_**S**_i_* in the direction of $\theta_{i}$ is

(86) $$W_{G_{i}S_{i}}=\tau_{i}-I_{i}\cdot {\ddot{\theta }}_{i}$$

The virtual displacements in each body of the mechanism must be compatible with the kinematic constraints by the joints. They must be related to a set of independent generalised virtual displacements that are served by $\delta q_{EE}$. Through Equations (8) and (10), the virtual displacements of the end effector can be expressed as

(87) $$\delta \chi_{EE}=\left[\begin{array}{c} M_{1b}\\ M_{0b} \end{array}\right]\cdot \delta q_{EE}$$

By Equations (20) and (24), the virtual displacements of the coupler ***S****_i_**M**_i_* are

(88) $$\delta \chi_{S_{i}M_{i}}=J_{S_{i}M_{i}}\cdot \delta q_{EE}$$

Finally, from Equation (65), the one-dimensional virtual displacement of the crank ***G****_i_**S**_i_* is related to $\delta q_{EE}$ as

(89) $$\delta \chi_{G_{i}S_{i}}=J_{\theta 1\left(i,:\right)}\cdot \delta q_{EE}$$

where $J_{\theta 1\left(i,:\right)}$ (*i* = 1, …, 6) is the *i*th row of $J_{\theta 1}$. The principle of virtual work for the inverse dynamics problem of the overall mechanism can be stated as

(90) $$\delta \chi_{EE}^{T}\cdot W_{EE}+\sum_{i=1}^{6}\delta \chi_{S_{i}M_{i}}^{T}\cdot W_{S_{i}M_{i}}+\sum_{i=1}^{6}\delta \chi_{G_{i}S_{i}}^{T}\cdot \left(\tau_{i}-I_{i}\cdot {\ddot{\theta }}_{i}\right)=0$$

where $W_{S_{i}M_{i}}$ is the resultant wrench acting at ***S****_i_**M**_i_*.

Substituting Equations (85)~(89) into Equation (90) and deleting the free term $\delta q_{EE}^{T}$, results in

(91) $$J_{\theta 1}^{T}\cdot \tau ={\left[\begin{array}{c} M_{1b}\\ M_{0b} \end{array}\right]}^{T}\cdot M_{3b}-\sum_{i=1}^{6}J_{S_{i}M_{i}}^{T}\cdot W_{S_{i}M_{i}}+J_{\theta 1}^{T}\cdot M_{9b}$$

which contains four equations and six unknowns, showing the mechanism is redundantly actuated.

### 6.2. Model 2

Analogous to the second model in Section 5.2, the dynamics model of the 6RSS PM is firstly built via the principle of virtual work, and next the two HKP constraints onto the effector are modelled. From Equations (67) and (81), the virtual displacements of the end effector and the crank ***G****_i_**S**_i_* are computed sequentially as

(92) $$\begin{array}{c} \delta \chi_{EE}=\left[\begin{array}{c} M_{1a}\\ M_{0a} \end{array}\right]\cdot \delta X_{EE}\\ \delta \chi_{G_{i}S_{i}}=J_{\theta 2\left(i,:\right)}\cdot \delta X_{EE} \end{array}$$

From Equations (20), (23) and (76), the virtual displacements of the *i*th coupler ***S****_i_**M**_i_* are

(93) $$\delta \chi_{S_{i}M_{i}}={\bar{J}}_{S_{i}M_{i}}\cdot \delta X_{EE}$$

Using the principle of virtual work, one can find that

(94) $$\delta \chi_{EE}^{T}\cdot W_{EE}+\sum_{i=1}^{6}\delta \chi_{S_{i}M_{i}}^{T}\cdot W_{S_{i}M_{i}}+\sum_{i=1}^{6}\delta \chi_{G_{i}S_{i}}^{T}\cdot \left(\tau_{i}-I_{i}\cdot {\ddot{\theta }}_{i}\right)=0$$

Upon substitution of Equations (85), (86), (92) and (93) into Equation (94), it results in

(95) $$\delta X_{EE}^{T}\cdot \left(-{\left[\begin{array}{c} M_{1a}\\ M_{0a} \end{array}\right]}^{T}\cdot M_{3b}+\sum_{i=1}^{6}{\bar{J}}_{S_{i}M_{i}}^{T}\cdot W_{S_{i}M_{i}}+J_{\theta 2}^{T}\cdot \tau -J_{\theta 2}^{T}\cdot M_{9b}\right)=0$$

Now the two HKP constraints are added to the end effector. From Equation (9), it yields that

(96) $$\delta X_{EE}=M_{J}\cdot \delta q_{EE}$$

Putting it into Equation (95) and omitting $\delta q_{EE}^{T}$ produces

(97) $$M_{J}^{T}\cdot \left({\left[\begin{array}{c} M_{1a}\\ M_{0a} \end{array}\right]}^{T}\cdot M_{3b}-\sum_{i=1}^{6}{\bar{J}}_{S_{i}M_{i}}^{T}\cdot W_{S_{i}M_{i}}+J_{\theta 2}^{T}\cdot M_{9b}\right)={\left(J_{\theta 2}\cdot M_{J}\right)}^{T}\cdot \tau$$

which completes the procedure with six unknowns and four equations. Identically, it also must be kept in mind that even though that all the terms in this equation are now functions of $q_{EE},{\dot{q}}_{EE},{\ddot{q}}_{EE}$ rather than $X_{EE},{\dot{X}}_{EE},{\ddot{X}}_{EE}$, the numerical values of the latter can be computed by the former and then fed into Equation (100), as illustrated in Section 5.2.2. Using the dynamics model of the 6RSS PM as the core can considerably alleviate the computational demands of the RAPM’s model, which will be exhibited in Section 7.4.3.

## 7. Numerical Computations and Comparisons

**Figure 7:**
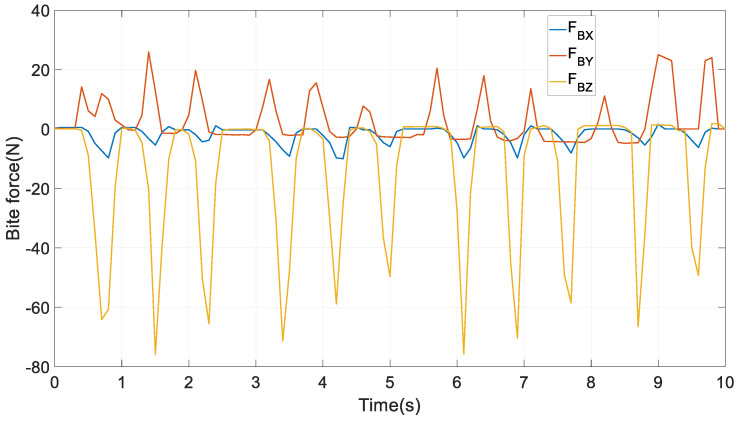
D bite force profiles on peanuts [4].

As an illustrative example, the mechanism is commanded to follow the first 5 s of a real incisor trajectory of a healthy human subject, as shown in Figure 6 of [36]. The trajectory viewed from different perspectives is given in the first four subplots in Figure 6 in this paper. The reason why only the first 5 s are adopted is, through the complete time interval of 10 s, the chewing trajectory almost experiences an analogous profile. Two interesting features are evident in these four subplots: firstly, from the 3D subplot, this trajectory is very arbitrary, compared with those in the pick-and-place manipulations in the industries as [37,38,39]. Secondly, the trajectory was concentrated as a straight line on the *X_S_*-*Z_S_* plane. The experimental setup and procedure to obtain the mastication movements in human subjects can be found in Chapter 6 of [4]. The corresponding mandibular motions in the four elements of ***q****_EE_* as a function of time are also provided in the following subplots, using the same method in Equation (38) of [2]: For the trajectory is defined along the three axes of {*S*}, only three scalar equations along these directions can be formulated; however, the RAPM has four DOFs, whereupon it is kinematically redundant to carry out this task, and one free DOF can be used to optimise the kinematic performance. In this regard, to minimise the two-norm sum of the tracking error is set as a simple optimal goal. In this process, the physical constraints imposed by the mechanism like the inverse kinematics equations and the workspace should be respected. Next, using Equation (6), the numerical values of *Z* and γ with respect to ***q****_EE_* can be computed. Finally, the first and second time derivatives of these six motion variables are also computed via Equations (9) and (83). From these subplots, one can see that the magnitude of *Z* is the largest in the translational displacements, so are $\dot{Z}$ and $\ddot{Z}$ in the translational velocities and accelerations, respectively. In terms of rotation, the angle *β* has a larger magnitude than both *α* and *γ*; correspondingly, $\dot{\beta }$ and $\ddot{\beta }$ are larger than $\dot{\alpha },\dot{\gamma }$ and $\ddot{\alpha },\ddot{\gamma }$, respectively. These exhibitions indicate that mouth opening/closing plays a dominant role when the lower jaw tracks the predefined trajectory. In tracking this prescribed chewing trajectory, neither the RAPM nor the 6RSS PM is at or near singular configurations. Correspondingly, the first 5 s of an experimentally measured 3D bite force in {*S*} on peanuts by a healthy human subject on the molars as in Figure 7, is exerted onto the right molar of the end effector. Numerical computations are performed to justify these models with a time step of 0.1 s. For the 6RSS PM without redundant actuations used as a benchmark in Section 7.4.2 and Section 7.4.4, its six DOFs and their first and second timederivatives as a function of time are identical to those in Figure 6. Correspondingly, the six chains in the two PMs undergo identical motions in terms of the numerical values of $q_{r_{i}},{\dot{q}}_{r_{i}},{\ddot{q}}_{r_{i}}\left(i=1,\ldots ,6\right)$.

### 7.1. Newton–Euler’s Approach

**Figure 8:**
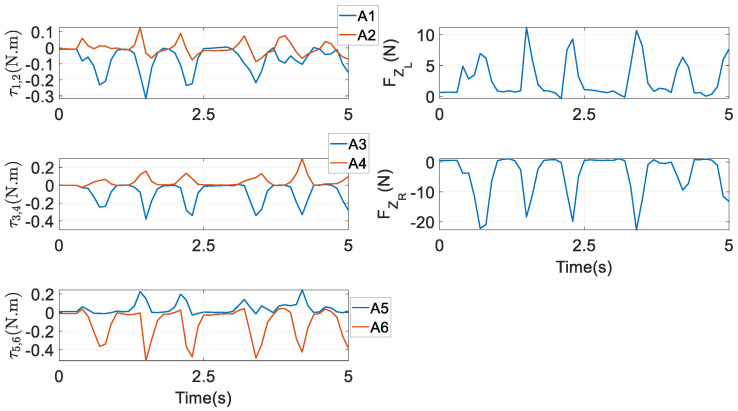
Actuating torques and constraint forces under Aim 1.

In this approach, the analytical expressions of the actuating torques τ, HKP constraint forces $F_{Z}$, and reaction forces at the spherical joints $F_{M_{1\_6}}$ and $F_{S_{1\_6}}$ all have been formulated. There are six equations and eight unknowns in Equation (38); thus, neither the torques nor the constrained forces can be determined uniquely or independently. Two aims related to the biomechanics of interest are set to optimally resolve the redundancy as

(98) $$\begin{array}{l} A_{1}=\min\left\|\tau \right\|\\ A_{2}=\min\left\|F_{Z}\right\| \end{array}$$

which correspond to the minimum efforts of the chewing muscles and minimum loads at TMJs, respectively. The following constraints

(99) $$\left\{\begin{array}{c} \left|\tau \right|\leq \tau_{\max}\\ the\;equality\;constraint\;in\;Eq.\;(38) \end{array}\right.$$

must be obeyed, where $\tau_{\max}$ is the maximum torque that can be generated by the actuators. The classical optimisation algorithm Sequential Quadratic Programming, which is characterised by its super-linear convergence, is adopted to address the problem. Under the first aim, the input torques and the constraint forces versus time along the chewing trajectory are exhibited in Figure 8. From it, the three pairs of actuators output almost symmetrical torques. Meanwhile, $F_{Z_{L}}$ and $F_{Z_{R}}$ are positive and negative, respectively, since the external reacted bite force are acting on a left molar in the end effector. Under the second objective they have an analogous profile, and thus are not exhibited for the sake of brevity. It is highlighted that after obtaining the constraint forces ***F****_Z_*, in view of Equations (37) and (38), the reaction forces at all the spherical joints can be computed with very modest additional computational demands.

Specifically, if $F_{Z}=0_{2\times 1}$ is assumed, which means *A*_2_ is ideally realised, Equation (38) has six equations with six unknowns in τ and they can be directly computed in a closed-form as

(100) $$\tau =M_{13b}$$

The physical meaning of Equation (100) is clear: when the torques satisfy this equation, there are no constraint forces from HKPs acting at the end effector. In this case, the torques are uniquely determined.

### 7.2. Lagrangian Formulation

In Equation (64), the only unknowns are the input torques can be optimised as

(101) $$\tau ={\left(J_{\theta 1}^{T}\right)}^{+}\cdot \left(M_{r1}\cdot {\ddot{q}}_{EE}+C_{r1}\cdot {\dot{q}}_{EE}+G_{r1}-F_{EE1}\right)$$

where ${\left(J_{\theta 1}^{T}\right)}^{+}$ is the pseudo-inverse of $J_{\theta 1}^{T}$, and its physical meaning is to minimize τ. While in the second model, the torques can also be minimised as

(102) $$\tau ={\left({\left(J_{\theta 2}\cdot M_{J}\right)}^{T}\right)}^{+}\cdot M_{J}^{T}\cdot \left(M_{r2}\cdot {\ddot{X}}_{EE}+C_{r2}\cdot {\dot{X}}_{EE}+G_{r2}-F_{EE2}\right)$$

### 7.3. Principle of Virtual Work

Analogous to the Lagrangian equation in Equation (64), the input torques from the two models based on the principle of virtual work can be minimised sequentially as

(103) $$\begin{array}{l} \tau ={\left(J_{\theta 1}^{T}\right)}^{+}\cdot \left({\left[\begin{array}{c} M_{1b}\\ M_{0b} \end{array}\right]}^{T}\cdot M_{3b}-\sum_{i=1}^{6}J_{S_{i}M_{i}}^{T}\cdot W_{S_{i}M_{i}}+J_{\theta 1}^{T}\cdot M_{21b}\right)\\ \tau ={\left({\left(J_{\theta 2}\cdot M_{J}\right)}^{T}\right)}^{+}\cdot M_{J}^{T}\cdot \left({\left[\begin{array}{c} M_{1a}\\ M_{0a} \end{array}\right]}^{T}\cdot M_{3b}-\sum_{i=1}^{6}{\bar{J}}_{S_{i}M_{i}}^{T}\cdot W_{S_{i}M_{i}}+J_{\theta 2}^{T}\cdot M_{21b}\right) \end{array}$$

### 7.4. Discussions

#### 7.4.1. Model Structures

The number of unknowns and equations in the inverse dynamics from different methods is summarised in Table 1, where NE means the model by Newton–Euler’s law, LA1 and LA2 means Model 1 and Model 2 formulated by the Lagrangian equations, PVW1 and PVW2 means Model 1 and Model 2 formulated by the principle of virtual work, respectively.

The similarity in this table is quite clear, i.e., there are always two unknowns over equations in the five models. Compared with the dynamics models of other RAPMs from the literature, it can also be found that the number of unknowns is always larger than that of equations. Specifically, in this RAPM at hand, from Newton–Euler’s law, there are two more constraint forces, while in the four models from the latter two methods, there are two more actuating torques.

However, the nature of this RAPM is only able to be clearly illustrated in Equation (38), which explicitly exhibits both the input torques and the HKP constraint forces. In other words, they must be optimised simultaneously in this model. Meanwhile, it is revealed that actuation redundancy is caused by the two HKP constraints: if ***F****_Z_* is directly set as zero in Equation (38), actuating torques can be directly obtained without optimisation.

In comparison, from the latter two methods, the information about the HKP constraint forces is not provided, and the only unknowns are input torques that can be computed directly. Nevertheless, the computation of ***F****_Z_* is critical: the pressure is high at both the condylar ball and the surface of the condylar socket due to the point contact. As a consequence, it is prone to cause wearing and then clearances. Meanwhile, the link attached by the condylar ball shown in Figure 2 is easily suffered from breaking if the constraint force is large. Apart from the fact that the number of unknowns is over equations from the four models, one cannot find the difference in the model structures between this RAPM at hand and others in publications. From these two aspects, the model from Newton–Euler’s law is superior to those from the latter two methods in revealing the nature of the RAPM.

Besides, determining how the number of the RSS kinematic chains varies the model structure is briefly presented in the following:

The end effector has 4 DOFs, thus it is assumed that there exist $n\left(n\geq 4\right)$ chains in the mechanism. In this regard, using Newton–Euler’s law in Section 4, $F_{M_{1\_n}}$ and $F_{S_{1\_n}}$ are in lieu of $F_{M_{1\_6}}$ and $F_{S_{1\_6}}$, respectively, and their sizes are both 3n×1; τ is a n×1 vector. The size of the matrices ***M***_2*b*_ in Equation (26), ***M***_5*b*_ and ***M***_6*b*_ in Equation (29), ***M***_7*b*_ in Equation (30), ***M***_8*b*_ and***M***_9*b*_ in Equation (34), are now 6×3n, 2n×3n, 2n×1, 3n×1, n×3n and n×1, sequentially. Equation (35) is now written as

(104) $${\left[\begin{array}{c} M_{2b}\\ M_{5b} \end{array}\right]}_{\left(6+2n\right)\times 3n}\cdot F_{M_{1\_n}}={\left[\begin{array}{c} M_{3b}\\ M_{6b} \end{array}\right]}_{\left(6+2n\right)\times 1}-{\left[\begin{array}{c} M_{4b}\\ 0_{2n\times 2} \end{array}\right]}_{\left(6+2n\right)\times 2}\cdot F_{Z}$$

It is easy to find that only when 6+2n=3n, i.e., *n* = 6, the precise values of $F_{M_{1\_n}}$ can be obtained as from Equation (36); otherwise, combining Equations (30), (34) and (35) gives rise to

(105) $$\left[\begin{array}{ccc} E_{n} & -M_{8b} & 0_{n\times 2}\\ 0_{\left(6+2n\right)\times n} & M_{15b} & M_{16b} \end{array}\right]\cdot \left[\begin{array}{c} \tau \\ F_{M_{1\_n}}\\ F_{Z} \end{array}\right]=\left[\begin{array}{c} M_{18b}\\ M_{17b} \end{array}\right]$$

where ***E****_n_* is the n×n identity matrix, and

$$M_{15b}=\left[\begin{array}{c} M_{2b}\\ M_{5b} \end{array}\right],\;M_{16b}=\left[\begin{array}{c} M_{4b}\\ 0_{2n\times 2} \end{array}\right],\;M_{17b}=\left[\begin{array}{c} M_{3b}\\ M_{6b} \end{array}\right],\;M_{18b}=M_{9b}+M_{8b}\cdot M_{7b}$$

From this process, the reaction forces at *M_i_* are now optimised together with τ and ***F****_Z_*: the number of kinematic chains only influences the existence of the reaction forces at *M_i_* while the HKP constraint forces always exist in the final dynamics equation Equation (105). In other words, the existence of HKP constraint forces ***F****_Z_* is independent of the number of kinematic chains in the final equation.

Proceeding in a like manner in the four models by the latter two methods, there would be four equations and *n* unknowns if the RAPM has *n* chains. The number of unknowns and equations is summarised in Table 2. From Netwon-Euler’s law, the number of unknowns is more than equations by $4n+2-\left(6+3n\right)=n-4$, as well as that from the latter two methods. Accordingly, Table 1 is just a summary of a specific case study compared with the generalised situation expressed in Table 2.

#### 7.4.2. Numerical Results

To justify and compare the five models numerically, two indices are set as

(106) $$F_{1}=\frac{1}{N}\sum_{i=1}^{N}\left\|\tau \right\|,F_{2}=\frac{1}{N}\sum_{i=1}^{N}\left\|F_{Z}\right\|$$

where *N* = 51 is the number of sampling points along the chewing trajectory. It is evident that *F*_2_ is only used specifically in the model via Newton–Euler’s law.

Their values under different optimal aims and models are listed in Table 3. One can find that *F*_1_ from the Newton–Euler approach under Aim 1, and the Lagrangian formulation and the principle of virtual work are identical. Thereby, it is sufficient to demonstrate the correctness and accuracy of the developed models. Furthermore, it is also noted that under Aim 2 from Newton–Euler’s law, the index *F*_2_ is nearly zero, and *F*_1_ is identical with four decimal places as its counterpart in the case when ***F****_Z_* is directly set to zero.

#### 7.4.3. Computational Cost

To assess the suitability of the models for real-time control, a reliable quantitative measure of the computational load is useful. The kinematic and dynamic parameters involved in the numerical computation can be found in [3]. The time consumption is summarised in Table 4. The procedures under each approach are all divided into symbolic and numeric computations, and have been implemented in programs written in MATLAB, using an Intel(R) Core(TM) i7-8700K CPU@3.70 GHz and 64 GB of RAM. Note that in LA2 and PVW2, the time for the numerical computation of $X_{EE},{\dot{X}}_{EE},{\ddot{X}}_{EE}$ by $q_{EE},{\dot{q}}_{EE},{\ddot{q}}_{EE}$ has been incorporated into the numerical time.

Under Newton–Euler’s law, the cost in the numeric computation for Aim 2 is nearly half of that for Aim 1, and it is almost equivalent to that when $F_{Z}=0_{2\times 1}$. Model 1 by the Lagrangian formulation is the most computationally intensive. The time consumption of Model 1 by the principle of virtual work is less than those via Newton–Euler’s law and the Lagrangian formulation. This discovery is in good agreement with the guess from [40]. The economy of computation for the RAPM seems to be quite remarkable.

By comparing the cost between the two models by the Lagrangian formulation, the latter one results in a considerably more economic algorithm for the solution of actuating torques, which is about 16.7% that of the first model. A similar comparison can be discovered between the two models under the principle of virtual work, though the second model is about 36.63% that of the first one. In addition, due to the fastest speed in the numerical computation in the second one, it has the potential to be readily employed in the model-based real-time motion and/or force control.

The main reason why the two second models from the energy methods are greatly more efficient is explained as follows: the resulting complexity of the two first models is a consequence of using the complex symbolic expressions of *Z* and γ in Equation (6), and the Jacobian matrix ***M****_J_*, for $q_{EE},{\dot{q}}_{EE},{\ddot{q}}_{EE}$ are used directly. By contrast, in the two second models, even though the matrices and vectors in Equations (84b) and (97) are ultimately functions of $q_{EE},{\dot{q}}_{EE},{\ddot{q}}_{EE}$, they can be computed by the numerical values of $X_{EE},{\dot{X}}_{EE},{\ddot{X}}_{EE}$ directly, which can be available from $q_{EE},{\dot{q}}_{EE},{\ddot{q}}_{EE}$ at the beginning of the formulation with a relatively minor cost, and then are fed into the left-hand side of Equations (84b) and (97). In this manner, the two second models in the latter two methods are free from the symbolic computation of *Z*, γ and ***M****_J_*, offering the simplest possible computational algorithms.

#### 7.4.4. Influence of HKP Constraints

The dynamics model of the 6RSS PM without HKPs is also built via the three above-mentioned methodologies, as far as the role of the HKP constraints in the numerical results and the computational cost is concerned. The modelling process is not listed for the sake of clarity. The 6RSS PM also tracks the predefined chewing trajectory as in Figure 6. It is found that based on these approaches, its inverse dynamics problem is reduced to solving a system of six linear equations in six unknowns. The input torques can be uniquely determined and there is no optimisation as in the RAPM. *F*_1_ always equals 0.2727 N∙m, which is also identical to that under Newton–Euler’s law when $F_{Z}=0_{2\times 1}$. The reason why this occurs is quite self-explanatory: the six DOFs and their first and second time derivatives in the 6RSS PM are numerically equivalent to $X_{EE},{\dot{X}}_{EE},{\ddot{X}}_{EE}$ of the RAPM. *F*_1_ is larger than those of the RAPM under Aim 1 from the three methods. It means that redundant actuation can minimise the input torques, which is a well-developed opinion and has been proved in a number of publications.

The computational time under the three methods is given in Table 5, from which one can also find that the computational burden of Newton–Euler’s law and the principle of virtual work is quite equivalent, and it is smaller than that from the Lagrange equations by a factor of about 2.2. By comparing Table 4 and Table 5, one can see that the two HKP constraints greatly increase the computational complexity. The computational cost in the RAPM under LA2 and PVW2, is quite equivalent to that in the 6RSS PM under the Lagrange equations and the principle of virtual work, respectively. It indicates that using the second models from the energy and virtual work related methods renders the computational demand comparable to that of the 6RSS PM. The findings above are self-explanatory: as the central feature in the two second models from the latter two methods, the core of the dynamics of the 6RSS PM has been employed in Equations (84b) and (97), and the computational time of the numerical $M_{J}^{T}$ is quite minor. As such, a computational very efficient formulation without accuracy deterioration in the dynamics model has been provided in Section 5.2 and Section 6.2.

From these comparisons, the HKP constraints greatly enhance the computational complexity in the model from Newton–Euler’s law and the two first models based on the energy and virtual work related methods, due to the complex expressions of *Z* and γ, and the Jacobian matrix $M_{J}$, etc.

## 8. Conclusions

The inverse dynamics of a spatial RAPM constrained by two HKPs was solved systematically in five models via the conventional Newton–Euler law, the Lagrangian equation, and the principle of virtual work. The scientific contribution of this paper is the deep study of inverse dynamics in a spatial RAPM with both lower kinematic pairs and HKPs, in terms of revealing the difficulties of HKP constraints. Specifically, the following conclusions can be drawn:

1. The robotic mechanism can satisfactorily imitate the chewing motions and forces of human beings. According to the computed actuating torques and constraint forces at spherical joints and HKPs, it is convenient to size actuators, linkages, HKP-related links in the mechanical design;
2. Under Newton–Euler’s law, the constraint forces from HKPs onto the end effector is revealed by this law quite well. In comparison, these forces do not appear in the models under the Lagrangian formulation and the principle of virtual work. The model by Newton–Euler’s law under the first objective aim and the four models from the latter two energy methods using the pseudo-inverse solution produce identical numerical results;
3. The efficiency of the model from Newton–Euler’s law and the two second models by the energy based methods is quite acceptable, while the first model by the Lagrangian formulation is extremely cumbersome. The two models from the principle of virtual work are the most computationally economic. In the two models by the Lagrangian formulations, the second one is much faster; an identical conclusion is also effective to the two models by the principle of virtual work. The computational cost from both the second models in the latter two methods approximately equals that of the 6RSS PM, mainly because the dynamics model of the 6RSS PM has been used as the core in these two second models;
4. By comparing the computational requirements between the RAPM and the 6RSS PM, it is discovered that the HKP constraints greatly raise the complexity of the mechanism.

## Figures and Tables

**Table 1:** Number of unknowns and equations.

	NE	LA1	LA2	PVW1	PVW2
Number of equations	6	4	4	4	4
Number of unknowns	8	6	6	6	6

**Table 2:** Number of unknowns and equations with $n\left(n\geq 4\right)$ kinematic chains.

	NE (*n* = 6)	$\mathrm{NE}\;\left(n\neq 6\right)$	LA1	LA2	PVW1	PVW2
Number of equations	6	6+3n	4	4	4	4
Number of unknowns	8	4n+2	*n*	*n*	*n*	*n*

**Table 3:** Indices from different models.

	NE			LA1	LA2	PVW1	PVW2
	Aim 1	Aim 2	$F_{Z}=0_{2\times 1}$				
*F*_1_ (N∙m)	0.1985	0.2727	0.2727	0.1985	0.1985	0.1985	0.1985
*F*_2_ (N)	7.7028	4.1477 × 10^−9^	-				

**Table 4:** Computational time of the RAPM (unit: s).

	NE			LA1	LA2	PVW1	PVW2
	Aim 1	Aim 2	$F_{Z}=0_{2\times 1}$				
Symbolic	9.720	9.720	9.720	39.054	7.731	9.923	4.311
Numeric	9.904	5.358	4.827	27.815	4.041	4.983	1.148
Total	19.624	15.078	14.547	66.869	11.772	14.906	5.459

**Table 5:** Computational time of the 6RSS PM (unit: s).

	NE	LA	PVW
Symbolic	4.074	7.716	4.071
Numeric	1.301	4.053	1.062
Total	5.375	11.769	5.133

## Data Availability

Data are available from the authors upon reasonable request.

## Nomenclature

The nomenclature of the primary variables is presented in the following table.

{*S*}	Inertia frame
{*M*}	Frame established at the mass centre *O_M_* of the end effector
*O_M_*	Mass centre of the end effector
α,β, and γ	Euler angles of the end effector
***G****_i_****S****_i_* (*i* = 1, …, 6)	The *i*th crank
***S****_i_****M****_i_* (*i* = 1, …, 6)	The *i*th coupler
{*C_i_*}	Frame established at the fixed point *G_i_* of the *i*th crank
{*N_i_*}	Frame attached at the mass centre *E_i_* of ***S****_i_**M**_i_*
*T_j_* (*j* = *L*, *R*)	Left and right condyle ball centres
$O_{S}O_{M}$	3 × 1 position vector of *O_M_* in {*S*}
*p_i_* (*i* = 1, …, 6)	Parameters describing the shape of surfaces where condyle ball centres *T_L_* and *T_R_* slide on
*X*, *Y*, and *Z*	Parameters describing translations of the end effector
RMS	Rotation matrix from {*S*} to {*M*}
$R_{X}\left(\alpha \right),R_{Y}\left(\beta \right)$, and $R_{Z}\left(\gamma \right)$	Rotation matrices about *X_M_*, *Y_M_*, and *Z_M_* axes by α,β, and γ, respectively
$X_{EE}$	6 × 1 vector grouping six motion variables of the end effector
${\dot{X}}_{EE},{\ddot{X}}_{EE}$	First and second time derivatives of $X_{EE}$, respectively
***q****_EE_*	4 × 1 vector grouping four DOFs of the end effector
${\dot{q}}_{EE},{\ddot{q}}_{EE}$	First and second time derivatives of ***q****_EE_*, respectively
$\omega_{EE}$	Angular velocity of the end effector
***M****_J_*	6 × 4 Jacobian matrix between ***X****_EE_* and ***q****_EE_*
$0_{3}$	3 × 3 zero matrix
***E***_3_	3 × 3 identity matrix
θ	6 × 1 vector containing the displacements of six active joints
$\left\|S_{i}M_{i}\right\|$ and $\left\|G_{i}S_{i}\right\|$	Length of ***S****_i_**M**_i_* and $G_{i}S_{i}$, respectively
$O_{M}M_{i}$ and OMMMi	Vectors of $O_{M}M_{i}$ in {*S*} and {*M*}, respectively
$O_{S}G_{i}$	3 × 1 constant position vector of *G_i_* in {*S*}
RNi0S and RCi0S	Rotation matrices from {*S*} to $\left\{N_{i}\right\}$ and $\left\{C_{i}\right\}$ at the initial configuration of the mechanism, respectively
$R_{Z}\left(\theta_{i}\right)$	Rotation matrix about the $Z_{C_{i}}$ axis by $\theta_{i}$
$\beta_{i}$ and $\gamma_{i}$	Euler angles expressing the rotation of the *i*th coupler
$q_{r_{i}},{\dot{q}}_{r_{i}},{\ddot{q}}_{r_{i}}\left(i=1,\ldots ,6\right)$	3 × 1 generalized vector specifying the configuration of the *i*th kinematic chain and its first and second time derivatives
$M_{1i}$	Jacobian matrix of $O_{S}G_{i}+G_{i}S_{i}+S_{i}M_{i}$ with respect to $q_{r_{i}}$
$M_{2i}$	Jacobian matrix of $O_{S}O_{M}+O_{M}M_{i}$ with respect to $q_{EE}$
$\omega_{S_{i}M_{i}}$ and ${\dot{\omega }}_{S_{i}M_{i}}$	Angular velocity and acceleration of ***S****_i_**M**_i_*, respectively
$V_{E_{i}}$ and ${\dot{V}}_{E_{i}}$	Translational velocity and acceleration of mass centre *E_i_* of ***S****_i_****M****_i_*, respectively
$J_{E_{i}}$	Jacobian matrix of $O_{S}E_{i}$ with respect to $q_{r_{i}}$
$F_{T_{i}}\left(i=L,R\right)$	Constrained forces at the left and right condylar balls, respectively
$F_{M_{i}}\left(i=1,\ldots ,6\right)$	Constrained forces at $M_{i}\left(i=1,\ldots ,6\right)$
$F_{S_{i}}\left(i=1,\ldots ,6\right)$	Constrained forces at $S_{i}\left(i=1,\ldots ,6\right)$
***g***	Gravitational acceleration vector
$F_{B}$	Bite force on the right molar
$F_{Z_{i}}\left(i=L,R\right)$	Magnitude of the constrained force at two HKPs along *Z_S_* axis
*m_EE_* and ***I****_EE_*	Mass and inertia matrix of the end effector, respectively
$m_{S_{i}M_{i}}$ and $I_{S_{i}M_{i}}$	Mass and the inertia matrix of ***S****_i_**M**_i_* with respect to *O_M_* and expressed in {*S*}, respectively
$I_{G_{i}S_{i}}$	Rotational inertia of ***G****_i_**S**_i_*
$\tau_{i}$	actuating torque provided by the *i*th actuator
τ	6 × 1 actuating torque vector by the six actuators
$T_{EE1},P_{EE1},L_{EE1}$	Kinetic energy, potential energy, and Lagrange function of the end effector in Model 1 under the Lagrangian equations
$M_{EE1},C_{EE1},G_{EE1},F_{EE1}$	4×4 mass matrix, 4×4 Coriolis and centrifugal force matrix, and 4×1 gravitational force vector, and the 4×1 generalised force vector of the end effector, respectively
$T_{S_{i}M_{i}},P_{S_{i}M_{i}}$	Kinetic energy and potential energy of the *i*th coupler
$T_{G_{i}S_{i}}$	Kinetic energy of the *i*th crank
$T_{G_{i}S_{i}M_{i}},P_{G_{i}S_{i}M_{i}},L_{G_{i}S_{i}M_{i}}$	Kinetic energy, the potential energy, and the Lagrangian function of the *i*th chain
$M_{G_{i}S_{i}M_{i}}\left(q_{r_{i}}\right)$, $C_{G_{i}S_{i}M_{i}}\left(q_{r_{i}},{\dot{q}}_{r_{i}}\right)$, $G_{G_{i}S_{i}M_{i}}\left(q_{r_{i}}\right)$, $F_{G_{i}S_{i}M_{i}}\left(\tau_{i}\right)$	3 × 3 mass matrix, 3 × 3 Coriolis/centrifugal force matrix, 3 × 1 gravitational force vector, and 3 × 1 generalized force vector of the *i*th chain, respectively
$q_{1}$	General coordinate vector in Model 1 by the Lagrangian formulation
$M_{1}\left(q_{1}\right)$, $C_{1}\left(q_{1},{\dot{q}}_{1}\right)$, $G_{1}\left(q_{1}\right)$, $F_{1}\left(\tau ,q_{EE}\right)$	Mass matrix, Coriolis and centrifugal force matrix, gravitational force vector, and generalised external force vector free of closed-loop constraints, respectively in Model 1
Φ	18 × 1 constraint equation vector
$\Phi_{q_{1}}$	18×22 constraint Jacobian matrix
$\lambda_{1}$ and $\lambda_{2}$	18×1 unknown Lagrangian multiplier vectors
$J_{\theta 1}$	6×4 Jacobian matrix between θ and $q_{EE}$
$T_{EE2},P_{EE2},L_{EE2}$	Kinetic energy, potential energy, and Lagrange function of the end effector in Model 2 under the Lagrangian equations
$M_{EE2},C_{EE2},G_{EE2},F_{EE2}$	6×6 mass matrix, 6×6 Coriolis and centrifugal force matrix, 6×1 gravitational force vector, and the 6×1 generalised force vector of the end effector, respectively, in Model 2
$q_{2}$	General coordinate vector in Model 2 by the Lagrangian formulation
$M_{2}\left(q_{2}\right)$, $C_{2}\left(q_{2},{\dot{q}}_{2}\right)$, $G_{2}\left(q_{2}\right)$, $F_{2}\left(\tau ,X_{EE}\right)$	Mass matrix, Coriolis and centrifugal force matrix, gravitational force vector, and generalised external force vector free of closed-loop constraints, respectively, in Model 2
$\Phi_{q_{2}}$	18×24 constraint Jacobian matrix
$J_{\theta 2}$	6×6 Jacobian matrix between θ and $X_{EE}$

## Appendix A

$$\begin{array}{l} M_{2b}={\left[\begin{array}{ccc} E_{3} & \cdots & E_{3}\\ O_{M}M_{1}\times & \cdots & O_{M}M_{6}\times \end{array}\right]}_{6\times 18}\;,\\ M_{3b}=\left[\begin{array}{c} m_{EE}\left({\dot{V}}_{O_{M}}+g\right)\\ I_{EE}\cdot {\dot{\omega }}_{EE}+\omega_{EE}\times \left(I_{EE}\cdot \omega_{EE}\right) \end{array}\right]-\left[\begin{array}{c} E_{3}\\ O_{M}B\times \end{array}\right]\cdot F_{B}\;,\;M_{4b}=\left[\begin{array}{cc} E_{3} & E_{3}\\ O_{M}T_{L}\times & O_{M}T_{R}\times \end{array}\right]\cdot \left(E_{2}⊗M_{H}\right)\;,\; \end{array}$$

where $I_{EE}$ is the 3×3 moment of inertia taken about the mass centre *O_M_*.

$$E_{S_{i}M_{i}}=I_{S_{i}M_{i}}\cdot {\dot{\omega }}_{S_{i}M_{i}}+\omega_{S_{i}M_{i}}\times \left(I_{S_{i}M_{i}}\cdot \omega_{S_{i}M_{i}}\right)+0.5\cdot m_{S_{i}M_{i}}\cdot S_{i}M_{i}\times \left({\dot{V}}_{E_{i}}+g\right)$$

$$M_{5b}={\left[\begin{array}{ccc} {\left(M_{1}S_{1}\times \right)}_{\left(1:2,:\right)} & & \\ & \ddots & \\ & & {\left(M_{6}S_{6}\times \right)}_{\left(1:2,:\right)} \end{array}\right]}_{12\times 18},M_{6b}={\left[\begin{array}{c} {E_{S_{1}M_{1}}}_{\left(1:2\right)}\\ \vdots \\ {E_{S_{6}M_{6}}}_{\left(1:2\right)} \end{array}\right]}_{12\times 1}$$

where ${\left(M_{i}S_{i}\times \right)}_{\left(1:2,:\right)}\left(i=1,\ldots ,6\right)$ denotes the first two rows of the skew-symmetric matrix $M_{i}S_{i}\times$, and ${E_{S_{i}M_{i}}}_{\left(1:2\right)}$ is a 2×1 vector containing the first two rows in $E_{S_{i}M_{i}}$.

$$M_{7b}={\left[\begin{array}{c} m_{S_{1}M_{1}}\cdot \left({\dot{V}}_{E_{1}}+g\right)\\ \vdots \\ m_{S_{6}M_{6}}\cdot \left({\dot{V}}_{E_{6}}+g\right) \end{array}\right]}_{18\times 1}$$

M8bi=GiCi0Si×3,:⋅RTCi0S,M8b=M8b1⋱M8b66×18

$$M_{9b}={\left[\begin{array}{c} I_{1}\cdot {\ddot{\theta }}_{1}\\ \vdots \\ I_{6}\cdot {\ddot{\theta }}_{6} \end{array}\right]}_{6\times 1}$$

$$M_{10b}={\left[\begin{array}{c} M_{2b}\\ M_{5b} \end{array}\right]}^{-1}\cdot \left[\begin{array}{c} M_{3b}\\ M_{6b} \end{array}\right]\;,\;M_{11b}={\left[\begin{array}{c} M_{2b}\\ M_{5b} \end{array}\right]}^{-1}\cdot \left[\begin{array}{c} M_{4b}\\ 0_{12\times 2} \end{array}\right]M_{12b}=M_{7b}+M_{10b}$$

$$\begin{array}{l} M_{13b}=M_{8b}\cdot M_{12b}+M_{9b}\\ M_{14b}=M_{8b}\cdot M_{11b} \end{array}$$

$$W_{S_{i}M_{i}}=-\left[\begin{array}{c} m_{S_{i}M_{i}}\cdot \left({\dot{V}}_{E_{i}}+g\right)\\ I_{S_{i}M_{i}}\cdot {\dot{\omega }}_{S_{i}M_{i}}+\omega_{S_{i}M_{i}}\times \left(I_{S_{i}M_{i}}\cdot \omega_{S_{i}M_{i}}\right) \end{array}\right]$$

JSiMi=JEiRNi0S⋅RωSiMi⋅M3i

J¯SiMi=JEiRNi0S⋅RωSiMi⋅M¯3i
